# Synergistic Effects of Mistletoe Lectin and Cisplatin on Triple-Negative Breast Cancer Cells: Insights from 2D and 3D In Vitro Models

**DOI:** 10.3390/ijms26010366

**Published:** 2025-01-03

**Authors:** Su-Yun Lyu, Saporie Melaku Meshesha, Chang-Eui Hong

**Affiliations:** 1 College of Pharmacy, Sunchon National University, Suncheon 57922, Republic of Korea; suyun96@yahoo.com (S.-Y.L.);; 2 Research Institute of Life and Pharmaceutical Sciences, Sunchon National University, Suncheon 57922, Republic of Korea

**Keywords:** triple-negative breast cancer, MDA-MB-231, Korean mistletoe lectin, cisplatin, synergistic effect, apoptosis, metastasis, 3D cell culture

## Abstract

Triple-negative breast cancer (TNBC) remains a challenging subtype due to its aggressive nature and limited treatment options. This study investigated the potential synergistic effects of Korean mistletoe lectin (*Viscum album* L. *var. coloratum* agglutinin, VCA) and cisplatin on MDA-MB-231 TNBC cells using both 2D and 3D culture models. In 2D cultures, the combination of VCA and cisplatin synergistically inhibited cell proliferation, induced apoptosis, and arrested the cell cycle at the G2/M phase. Also, the combination treatment significantly reduced cell migration and invasion. Gene expression analysis showed significant changes in specific genes related to apoptosis (Bax, Bcl-2), metastasis (MMP-2, MMP-9), and EMT (E-cadherin, N-cadherin). Three-dimensional spheroid models corroborated these findings, demonstrating enhanced cytotoxicity and reduced invasion with the combination treatment. Significantly, the 3D models exhibited differential drug sensitivity compared to 2D cultures, emphasizing the importance of utilizing physiologically relevant models in preclinical studies. The combination treatment also reduced the expression of angiogenesis-related factors VEGF-A and HIF-1α. This comprehensive study provides substantial evidence for the potential of VCA and cisplatin combination therapy in TNBC treatment and underscores the significance of integrating 2D and 3D models in preclinical cancer research.

## 1. Introduction

Breast cancer is one of the most common cancers among women. The Global Cancer Observatory (GLOBOCAN) reported in 2022 that there were 2.3 million breast cancer cases and 666,000 deaths from this disease [1]. Advanced or metastatic stages remain difficult to treat even with current detection and treatment methods [2]. Although the five-year survival rate for localized breast cancer is 99%, once it spreads, only 27% of people survive for five years [3]. This poor survival rate stems from the development of drug resistance to various treatments, including chemotherapy, hormone therapy, and targeted drugs [4]. The resistance mechanisms include enhanced cell survival pathways, altered drug transport and metabolism, and increased DNA repair capacity [5,6]. Overcoming these resistance mechanisms is crucial for improving patient outcomes [7].

Compared to other types of breast cancer, triple-negative breast cancer (TNBC) is known for being particularly difficult to treat and affects outcomes differently [8]. TNBC is characterized by the lack of three key receptors: estrogen receptor (ER), progesterone receptor (PR), and human epidermal growth factor receptor 2 (HER2) [9]. Due to this molecular profile, 15% to 20% of breast cancer patients with TNBC do not respond to conventional targeted therapies such as tamoxifen, aromatase inhibitors, or trastuzumab [10]. Therefore, despite its drawbacks and potential side effects, chemotherapy remains a primary treatment method for addressing TNBC [11]. TNBC is more common in women under the age of 50 and is prevalent among African, Hispanic, and Asian women [12,13], suggesting potential genetic and environmental factors in its development [14,15]. TNBC is often diagnosed at a more advanced stage with larger tumors and higher grades compared to other breast cancer subtypes [16]. This aggressive nature leads to a poor prognosis for TNBC patients, who may face rapid disease progression, early relapse, and an increased risk of distant metastasis to areas like the lungs, brain, and bones [17,18]. Due to the heterogeneity in TNBC, it is challenging to treat and manage the condition [19]. Recent advancements in the field of biology and pharmaceutical research have led to the identification of treatment targets and innovative strategies for TNBC [20]. For instance, drugs like Olaparib and talazoparib, which are poly (ADP-ribose) polymerase (PARP) inhibitors, have shown good results in TNBC patients who have inherited BRCA1/2 mutations [21,22]. Moreover, immune checkpoint inhibitors, such as atezolizumab and pembrolizumab, have shown effectiveness in a subgroup of TNCB patients with high levels of programmed death-ligand 1 (PD-L1) expression [22,23]. Researchers are also investigating targets such as androgen receptors, epidermal growth factor receptor (EGFR), and vascular endothelial growth factor (VEGF) [24,25].

Given the challenges in treating TNBC and the need for more effective therapies, researchers have turned to well-characterized cell line models to study the disease mechanisms and test potential treatments. Among these, MDA-MB-231 is one of the most commonly employed cell lines derived from the pleural effusion of a 51-year-old Caucasian female patient with TNBC [26]. Established in 1973, this cell line has been instrumental in elucidating the molecular events that contribute to the emergence, progression, and chemoresistance of TNBC [27]. MDA-MB-231 cells are particularly valuable for TNBC research due to their aggressive and highly invasive nature, which closely mimics the challenging clinical characteristics of TNBC [28]. MDA-MB-231 cells demonstrate aggressive metastatic properties, characterized by enhanced motility and invasiveness compared to other breast cancer cell lines. This invasive capacity stems from elevated matrix metalloproteinases (MMPs), particularly MMP-2 and MMP-9, which facilitate extracellular matrix degradation [29]. The cells express high levels of chemokine receptors CXCR4 and CCR7, enabling metastasis to the lungs, bones, and brain [30,31]. Their aggressive phenotype is further enhanced by the epithelial-to-mesenchymal transition (EMT), which confers stem cell-like properties [32]. During the EMT, epithelial markers, such as E-cadherin, are downregulated, while mesenchymal markers, such as vimentin and N-cadherin, are upregulated [33]. This process is regulated by transcription factors, i.e., Snail, Slug, and Twist, which are over-expressed in MDA-MB-231 cells [34]. These cells are more invasive, metastatic, and resistant to apoptosis through the activation of EMT programs [35].

Traditional two-dimensional (2D) cell culture models using MDA-MB-231 cells have been widely used in TNBC research. However, these models often fail to recapitulate the complex three-dimensional (3D) environment, cell–cell interactions, and drug penetration barriers observed in solid tumors [36]. Three-dimensional cultures, such as multicellular spheroids and organoids, better simulate important physiological features, including (1) oxygen and nutrient gradients that affect drug distribution, (2) cell–cell and cell–matrix interactions that influence drug response, (3) extracellular matrix organization that impacts drug penetration, and (4) cellular heterogeneity often observed in patient tumors [37]. Recent studies have demonstrated that these 3D models, particularly patient-derived organoids, can more accurately predict chemotherapy efficacy and resistance patterns compared to traditional 2D cultures [38]. For instance, drug penetration barriers observed in 3D models have revealed mechanisms of resistance not detected in 2D systems [39]. The integration of both 2D and 3D models in preclinical studies can, therefore, provide more comprehensive insights into drug efficacy and mechanisms of action [40]. This approach is particularly relevant for TNBC research, where the development of effective therapies remains a significant challenge [41]. By utilizing both culture systems, researchers can gain insights into drug effects at different levels of complexity, potentially leading to more translatable findings for clinical applications [42].

Cisplatin, a platinum-based compound, enters the cell and binds to DNA, causing DNA damage such as breaks and cross-linking that prevent cell division and lead to cell death [43]. However, the efficacy of cisplatin against MDA-MB-231 cells is comparatively low because of the emergence of drug resistance mechanisms [44]. These resistance mechanisms include enhanced drug efflux through P-glycoprotein (P-gp) and breast cancer resistance protein (BCRP) [45], increased DNA repair capacity [46], altered apoptotic signaling [47], and a modified tumor microenvironment [48]. These cells serve as a valuable model for studying cisplatin resistance and developing combination therapeutic strategies [49]. When cisplatin is combined with other chemotherapy agents like paclitaxel or doxorubicin, antitumor efficacy is improved in animal models [50,51]. Additionally, targeted agents such as PARP inhibitors and antiangiogenic agents have been suggested to enhance the sensitivity of MDA-MB-231 cells to cisplatin [52,53]. Another strategy to modulate cisplatin resistance in MDA-MB-231 cells involves the use of natural compounds with chemopreventive or chemosensitizing activities [54]. For instance, curcumin and resveratrol have been shown to increase the cytotoxic effect of cisplatin on MDA-MB-231 cells by modifying biological activities [55,56].

*Viscum album* L. var. *coloratum* agglutinin (VCA) belongs to a unique class of type II ribosome-inactivating proteins (RIPs) that exhibit both N-glycosidase and carbohydrate-binding activities [57,58,59]. Compared to European mistletoe lectin, VCA demonstrates enhanced ribosome-inactivating activity and cytotoxicity against cancer cells, attributed to its distinct glycan profile that enables selective cellular uptake [57,60]. This cancer-specific cytotoxicity extends to various malignancies, including breast, lung, and colon cancers, while sparing normal cells [61]. The selective targeting stems from the differential expression of carbohydrate receptors between cancer and normal cells [60]. At the molecular level, VCA induces apoptosis through multiple pathways, including the activation of caspase cascades, modulation of Bcl-2 family proteins, and generation of reactive oxygen species [62,63]. Studies have shown that VCA specifically targets cancer cells through the recognition of altered glycosylation patterns on their surfaces, a characteristic feature of malignant transformation [64]. Furthermore, VCA has a strong antiangiogenic effect, which suppresses the formation of new blood vessels, as well as the level of vascular endothelial growth factor (VEGF) in tumor tissues [60]. VCA also has immunomodulatory properties, increasing the function of NK cells and possibly triggering the host’s immune system to identify and destroy cancerous cells [65].

Several clinical studies have explored the use of mistletoe extract as an adjuvant to standard chemotherapeutic drugs in different kinds of cancer. These studies have described enhanced results that have highlighted increased quality of life, fewer side effects, and increased survival time [66,67]. The rationale for exploring the VCA–cisplatin combination is particularly compelling, as these agents may act through complementary mechanisms. While cisplatin primarily induces DNA damage and cell cycle arrest [68], VCA’s multi-targeted approach, including protein synthesis inhibition and immunomodulation [69], could potentially enhance therapeutic efficacy while minimizing drug resistance [70]. Recent studies have explored various combination strategies for TNBC, including PARP inhibitors with platinum agents (e.g., olaparib) and immunotherapy combinations (e.g., pembrolizumab) [22,23]. However, these approaches often face challenges of toxicity or limited efficacy in heterogeneous TNBC populations. The VCA–cisplatin combination offers unique advantages through its dual targeting of DNA damage and protein synthesis pathways, potentially addressing multiple resistance mechanisms simultaneously. Preclinical studies support this approach, with VCA showing synergistic effects when combined with conventional chemotherapeutics [71] while modulating the tumor microenvironment in breast cancer cells [72].

Additionally, VCA’s selective toxicity towards cancer cells might help reduce the overall cytotoxic burden of combination therapy [73]. However, the molecular mechanisms underlying the potential synergistic effects of VCA and conventional chemotherapy, particularly in aggressive cancers like TNBC, remain poorly understood [74,75]. This knowledge gap is particularly significant given the urgent need for more effective treatment strategies for TNBC, where conventional therapies often show limited efficacy due to drug resistance and heterogeneity [76]. Therefore, the specific effects of mistletoe lectin in combination with cisplatin on TNBC cells, particularly the highly aggressive and metastatic MDA-MB-231 cell line, have not been thoroughly investigated.

The current work aims to investigate the potential synergistic effects of VCA and cisplatin in both 2D and 3D models of TNBC using MDA-MB-231 cells, employing a comprehensive approach that includes cytotoxicity assays, migration and invasion studies, gene and protein expression analyses, angiogenesis factor evaluation, and 3D spheroid characterization. This study seeks to provide a more complete understanding of the combination therapy’s efficacy and mechanisms of action, potentially offering new insights into the management of this aggressive cancer type.

## 2. Results

### 2.1. Dose-Dependent Cell Viability Response in 2D Culture 

**Figure 1:**
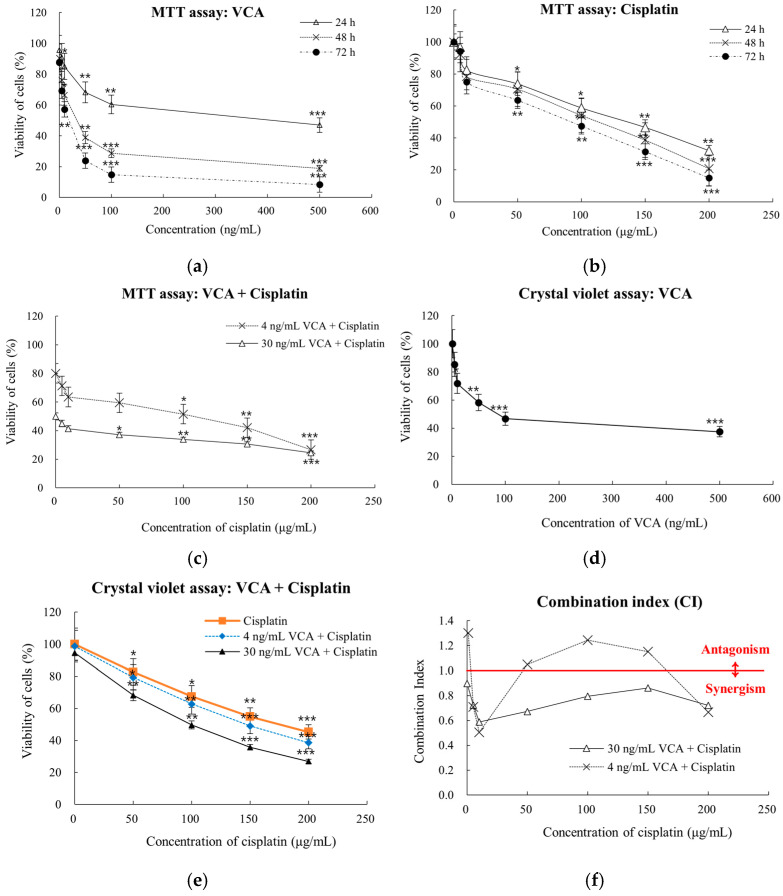

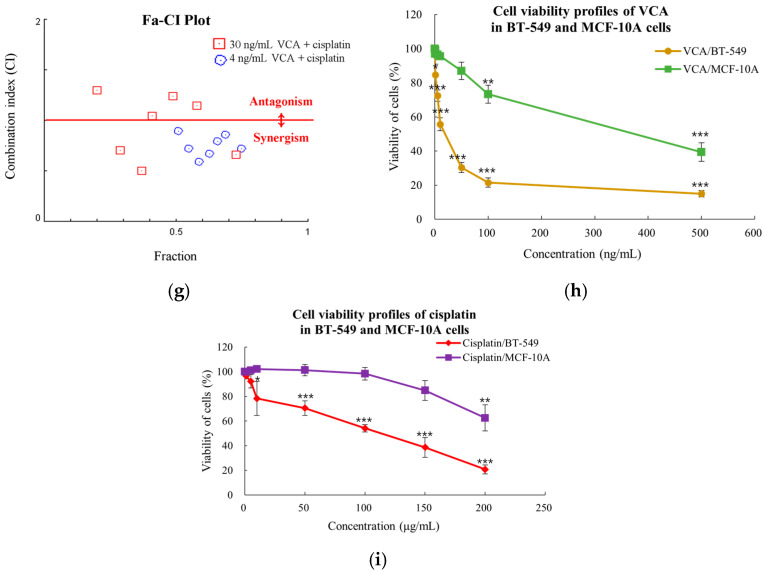
Cytotoxic effects of VCA and cisplatin on MDA-MB-231 and MCF-10A cells. Cells were treated with (**a**) VCA (1–500 ng/mL), (**b**) cisplatin (5–200 μg/mL), or (**c**) VCA plus cisplatin for 24, 48, and 72 h, and viability was measured by the MTT assay in MDA-MB-231 cells. Crystal violet assay results for (**d**) VCA, (**e**) cisplatin, and their combination after 48 h treatment in MDA-MB-231 cells. (**f**) Combination index (CI) and (**g**) fractional effect (Fa) were obtained by the non-constant ratio combination method. For comparison, BT-549 TNBC-derived cells and MCF-10A normal breast epithelial cells were treated with (**h**) VCA (1–500 ng/mL) or (**i**) cisplatin (5–200 μg/mL) for 48 h, and cell viability was assessed by the WST-1 assay. Data are presented as mean ± SD with 95% confidence intervals from three independent experiments. Statistical significance was determined using one-way ANOVA followed by Dunnett’s post hoc test for single treatments and two-way ANOVA followed by Tukey’s post hoc test for combinations. * *p* < 0.05, ** *p* < 0.01, *** *p* < 0.001 compared to the untreated control (one-way ANOVA followed by Dunnett’s post hoc test for single treatments; two-way ANOVA followed by Tukey’s post hoc test for combinations).

The effects of VCA and cisplatin on MDA-MB-231 cell viability were assessed using both MTT and crystal violet assays. The MTT assay results showed that VCA treatment for 24, 48, and 72 h led to a dose-dependent decrease in cell viability with 95% confidence intervals (Figure 1a). All experiments were performed in three independent replicates. Significant reductions in viability were observed at concentrations as low as 50 ng/mL after 24 h (*p* < 0.05), with more significant effects at higher concentrations and longer exposure times. At 24 h, significant reductions in viability were observed starting at 10 ng/mL VCA (85.01%, *p* < 0.05), with more pronounced effects at higher concentrations (47.01% viability at 500 ng/mL, *p* < 0.001). Similarly, cisplatin treatment resulted in dose-dependent cytotoxicity, with significant effects observed at 10 μg/mL (82.45%, *p* < 0.05), reaching maximum inhibition at 200 μg/mL (32.04% viability) (Figure 1b). The combination of VCA and cisplatin demonstrated enhanced cytotoxic effects compared to either agent alone (Figure 1c). At 48 h, two combination strategies were evaluated: low-dose (4 ng/mL VCA) and high-dose (30 ng/mL VCA) combined with various cisplatin concentrations. The high-dose combination showed stronger effects, reducing cell viability to 24.58% at 200 μg/mL cisplatin (*p* < 0.001) compared to 26.68% for the low-dose combination.

To validate the MTT assay results and address potential mitochondrial dysfunction, a crystal violet assay was conducted at the 48 h time point (Figure 1d,e). The crystal violet assay confirmed the dose-dependent cytotoxic effects of VCA and cisplatin, showing significant viability reductions at 10 ng/mL VCA (71.8%, *p* < 0.05) and 50 μg/mL cisplatin (82.6%, *p* < 0.05). The combination treatments showed enhanced effects, with the 30 ng/mL VCA combination reducing viability to 26.9% at 200 μg/mL cisplatin. While the absolute viability percentages were slightly higher in the crystal violet assay, the overall trends and relative differences between treatments were consistent with the MTT results. Significant reductions in viability were observed for VCA concentrations of 10 ng/mL and above (*p* < 0.05 to *p* < 0.001) and for cisplatin concentrations of 50 μg/mL and above (*p* < 0.05 to *p* < 0.01). Combination index (CI) analysis demonstrated synergistic interactions at specific concentration ranges (Figure 1f,g). The 30 ng/mL VCA combination exhibited strong synergy with 10 μg/mL cisplatin (CI = 0.588), while the 4 ng/mL VCA combination showed synergy with 10 μg/mL cisplatin (CI = 0.504).

To confirm these effects in another TNBC cell line, we tested VCA and cisplatin on BT-549 cells. The results showed similar dose-dependent cytotoxicity patterns. Statistical analysis revealed significant decreases in cell viability with VCA treatment starting at 1 ng/mL (*p* < 0.05), becoming more pronounced at 5 ng/mL and above (*p* < 0.001). Cisplatin showed significant effects from 10 μg/mL (*p* < 0.05), with stronger effects at 50 μg/mL and above (*p* < 0.001). The IC values for BT-549 cells (IC20 = 7.2 ng/mL, IC50 = 23.5 ng/mL for VCA; IC20 = 82 μg/mL, IC50 = 143 μg/mL for cisplatin) were comparable to those of MDA-MB-231 cells, confirming the consistency in drug effects across different TNBC cell lines (Figure 1h,i). These findings suggest that both VCA and cisplatin demonstrate preferential cytotoxicity towards breast cancer cells while showing relatively lower toxicity to normal breast epithelial cells.

### 2.2. Flow Cytometric Analysis and Live/Dead Cell Assay for Apoptosis in 2D Cells

**Figure 2:**
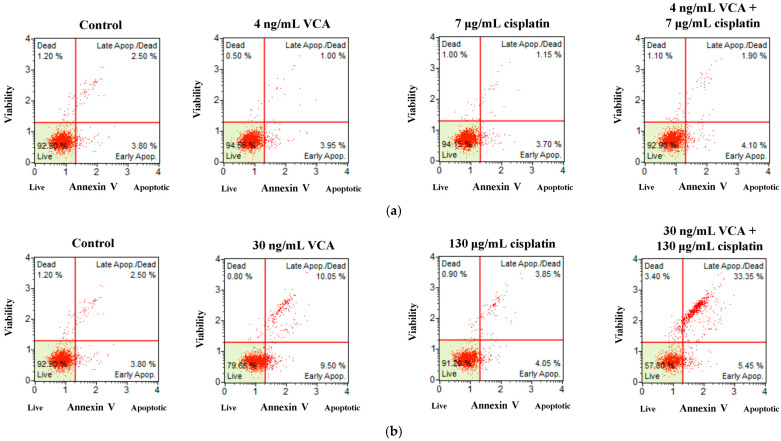

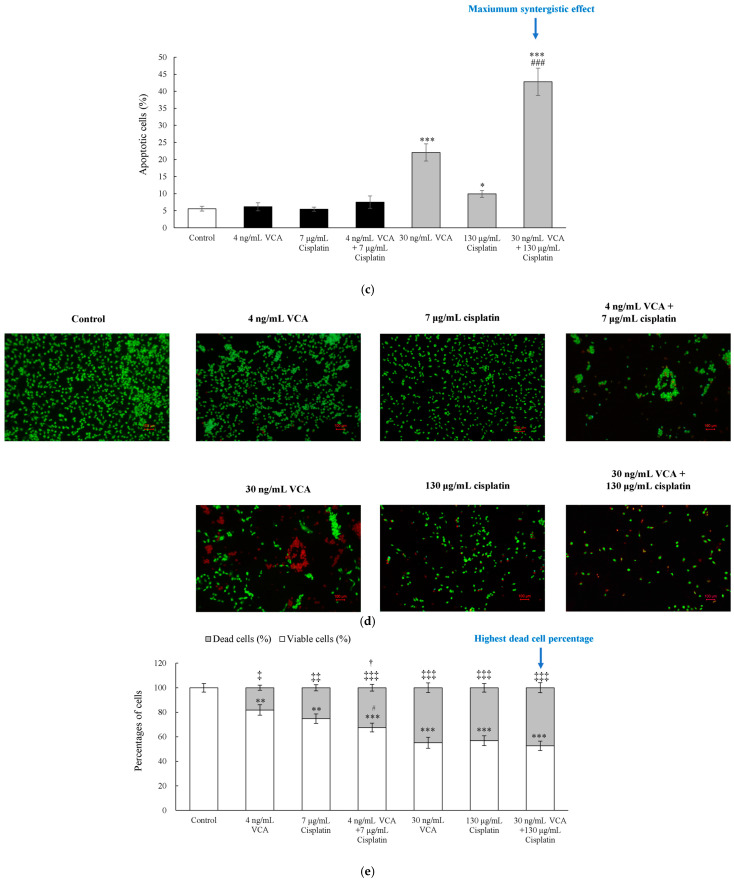
Analysis of apoptosis in MDA-MB231 cells. (**a**) Flow cytometric analysis of cells treated with 4 ng/mL VCA (IC20), 7 μg/mL cisplatin (IC20), or their combination for 48 h. (**b**) Flow cytometric analysis of cells treated with 30 ng/mL VCA (IC50), 130 μg/mL cisplatin (IC50), or their combination for 48 h. Cells were stained with Annexin V-FITC and PI. (**c**) Quantification of apoptotic cells (early + late apoptosis) from flow cytometry data based on ImageJ analysis version 1.54m. Data are presented as mean ± SD from three independent experiments. Statistical significance was determined using one-way ANOVA followed by Tukey’s post hoc test. * *p* < 0.05, *** *p* < 0.001 compared to control; ### *p* < 0.001 compared to respective single treatments. (**d**) Representative fluorescence microscopy images of MDA-MB-231 cells treated with VCA (4 or 30 ng/mL), cisplatin (7 or 130 μg/mL), or their combinations for 48 h. Images shown are representative of the multiple fields examined; quantification was performed across multiple fields from three independent experiments. While individual representative images may show varying distributions of live/dead cells, the quantitative data represents the average values obtained from a systematic analysis of multiple fields. Cells were stained with acridine orange (green, live cells) and propidium iodide (red, dead cells). Scale bar = 100 μm. (**e**) Quantification of viable and dead cells based on ImageJ analysis of live/dead staining. Data are presented as mean ± SD with 95% confidence intervals from three independent experiments, with at least 10,000 events analyzed per sample. Statistical significance was determined using one-way ANOVA followed by Tukey’s post hoc test. For viable cells: ** *p* < 0.01, *** *p* < 0.001 compared to control; # *p* < 0.05 compared to respective single treatments. For dead cells: ‡ *p* < 0.05, ‡‡ *p* < 0.01, ‡‡‡ *p* < 0.001 compared to control; † *p* < 0.05 compared to respective single treatments.

We investigated whether the synergistic effect of VCA and cisplatin on cell viability resulted from increased apoptosis. MDA-MB-231 cells were treated with VCA (4 ng/mL or 30 ng/mL), cisplatin (7 μg/mL or 130 μg/mL), or their combination for 48 h, corresponding to IC20 and IC50 levels. We analyzed apoptosis using Annexin V and PI staining with flow cytometry. Quantitative analysis demonstrated that the combination of VCA and cisplatin significantly increased apoptotic cell percentages compared to individual treatments (Figure 2a–c). The combination of 30 ng/mL VCA and 130 μg/mL cisplatin induced apoptosis in 42.8 ± 4.0% of cells with 95% confidence intervals, based on three independent experiments. This was significantly higher than VCA (22.05 ± 2.5%, *p* < 0.01) or cisplatin (9.9 ± 1.0%, *p* < 0.001) alone. Similarly, the lower dose combination (4 ng/mL VCA + 7 μg/mL cisplatin) induced 7.5 ± 1.8% apoptosis compared to VCA (6.15 ± 1.2%) or cisplatin (5.45 ± 0.6%) alone (*p* > 0.05). To validate these apoptotic effects, cell viability was assessed using acridine orange (AO) and propidium iodide (PI) staining (Figure 2d). A quantitative analysis was performed using standardized ImageJ protocols across multiple fields from three independent experiments. The control cells exhibited mainly green fluorescence, indicating viability. Treatment with 30 ng/mL VCA or 130 μg/mL cisplatin alone for 48 h increased the number of red-fluorescent dead cells and decreased green-fluorescent live cells. The combination treatment further enhanced this effect. Merged images revealed a shift from mostly live cells in controls to increased dead cells in the combination treatment. The quantification results showed that treatment with VCA and cisplatin, alone and in combination, significantly affected the viability of MDA-MB-231 cells (Figure 2e). Compared to the control, both VCA and cisplatin individually reduced cell viability in a dose-dependent manner. At lower concentrations, 4 ng/mL VCA and 7 μg/mL cisplatin decreased viable cells to 81.85% (*p* < 0.01) and 74.83% (*p* < 0.01), respectively, while increasing dead cells to 18.15% (*p* < 0.05) and 25.17% (*p* < 0.01), respectively. Higher concentrations of 30 ng/mL VCA and 130 μg/mL cisplatin further reduced viability to 55.09% (*p* < 0.001) and 56.82% (*p* < 0.001), respectively, with corresponding increases in dead cells to 44.91% (*p* < 0.001) and 43.18% (*p* < 0.001). Notably, the combination treatments showed enhanced effects. The lower dose combination (4 ng/mL VCA + 7 μg/mL cisplatin) reduced viability to 67.49% (*p* < 0.001 vs. control, *p* < 0.05 vs. individual treatments) and increased dead cells to 32.51% (*p* < 0.001 vs. control, *p* < 0.05 vs. individual treatments). The higher dose combination (30 ng/mL VCA + 130 μg/mL cisplatin) showed the most significant effect, reducing viability to 52.63% (*p* < 0.001 vs. control, *p* < 0.01 vs. 30 ng/mL VCA, *p* < 0.05 vs. 130 μg/mL cisplatin) and increasing dead cells to 47.37% (*p* < 0.001 vs. control, *p* < 0.01 vs. 30 ng/mL VCA, *p* < 0.05 vs. 130 μg/mL cisplatin).

### 2.3. Two-Dimensional Cell Cycle Analysis

**Figure 3:**
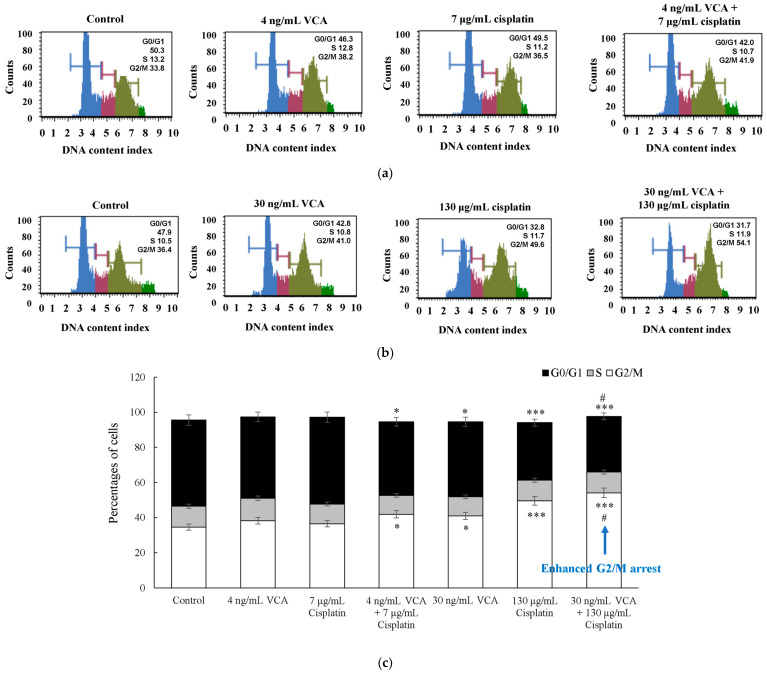
Cell cycle analysis of MDA-MB231 cells. (**a**) Cells were treated with 4 ng/mL VCA (IC20), 7 μg/mL cisplatin (IC20), or their combination for 48 h. (**b**) Cells were treated with 30 ng/mL VCA (IC50), 130 μg/mL cisplatin (IC50), or their combination for 48 h. Cells were fixed, stained with PI, and analyzed by flow cytometry. Histograms show the distribution of cells in different phases of the cell cycle. (**c**) Quantification of cells in the G0/G1, S, and G2/M phases. Data are presented as mean ± SD with 95% confidence intervals from three independent experiments, with at least 10,000 events analyzed per sample. Statistical significance was determined using one-way ANOVA followed by Tukey’s post hoc test. * *p* < 0.05, *** *p* < 0.001 compared to control; # *p* < 0.05 compared to respective single treatments.

The effects of VCA and cisplatin on MDA-MB-231 cell cycle progression were examined using PI staining and flow cytometry. PI fluorescence intensity directly correlates with DNA content, allowing us to determine the percentage of cells in each phase of the cell cycle (G0/G1, S, and G2/M). Cells were exposed to 4 ng/mL VCA (IC20), 7 μg/mL cisplatin (IC20), or both for 48 h; then, their cell cycle phases were measured. As shown in Figure 3a–c, the cell cycle analysis demonstrated that both VCA and cisplatin, alone and in combination, induced G2/M phase arrest. Based on three independent experiments with 95% confidence intervals, in control cells, the G0/G1, S, and G2/M phase distributions were 49.1 ± 3.0%, 11.85 ± 1.5%, and 34.6 ± 2.5%, respectively. Low-dose treatments with either 4 ng/mL VCA (G0/G1: 46.3 ± 2.8%, S: 12.8 ± 1.6%, G2/M: 38.2 ± 2.8%) or 7 μg/mL cisplatin (G0/G1: 49.5 ± 3.1%, S: 11.2 ± 1.4%, G2/M: 36.5 ± 2.7%) showed minimal effects on the cell cycle distribution. Their combination resulted in a moderate increase in the G2/M phase (41.9 ± 3.0%, *p* < 0.05) with a corresponding decrease in the G0/G1 phase (42.0 ± 2.5%, *p* < 0.05). High-dose treatments had more pronounced effects: 30 ng/mL VCA increased G2/M phase populations to 41.0 ± 3.0% (*p* < 0.05), and 130 μg/mL cisplatin increased them to 49.6 ± 3.5% (*p* < 0.001). The combination of 30 ng/mL VCA and 130 μg/mL cisplatin showed the strongest effect, with the G2/M phase reaching 54.1 ± 3.8% (*p* < 0.001 vs. control, *p* < 0.05 vs. single treatments) and the G0/G1 phase decreasing to 31.7 ± 1.9% (*p* < 0.001 vs. control, *p* < 0.05 vs. single treatments). The S phase percentages showed minimal variation across treatments (ranging from 10.7% to 12.8%). These results indicate VCA and cisplatin’s growth-inhibitory effects may partly stem from G2/M phase arrest, impeding cell cycle progression. 

### 2.4. Transwell Migration and Invasion Assays in 2D Cells

**Figure 4:**
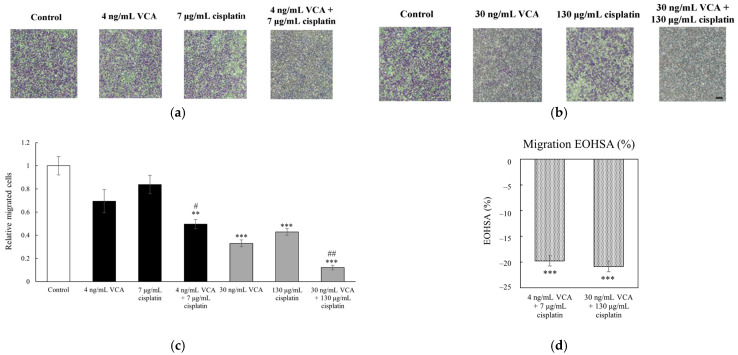
Effects of VCA and cisplatin on cell migration in MDA-MB-231 cells. Cells were treated with (**a**) 4 ng/mL VCA (IC20) or 7 μg/mL cisplatin (IC20) or 4 ng/mL VCA plus 7 μg/mL cisplatin and (**b**) 30 ng/mL VCA (IC50) or 130 μg/mL cisplatin (IC50) or their combinations for 12 h. Migrated cells were observed using a light microscope (magnification, ×40; scale bar, 100 μm). (**c**) The number of migrated cells was quantified using ImageJ software. Data are presented as relative migrated cells compared to control, with error bars representing standard deviation from three independent experiments. (**d**) Excess Over Highest Single Agent (EOHSA) analysis of combination treatments. EOHSA values below 0 indicate synergistic effects. Data are presented as mean ± SD with 95% confidence intervals from three independent experiments. Statistical significance was determined using one-way ANOVA followed by Tukey’s post hoc test. ** *p* < 0.01, *** *p* < 0.001 compared to control; # *p* < 0.05, ## *p* < 0.01 compared to respective single treatments.

**Figure 5:**
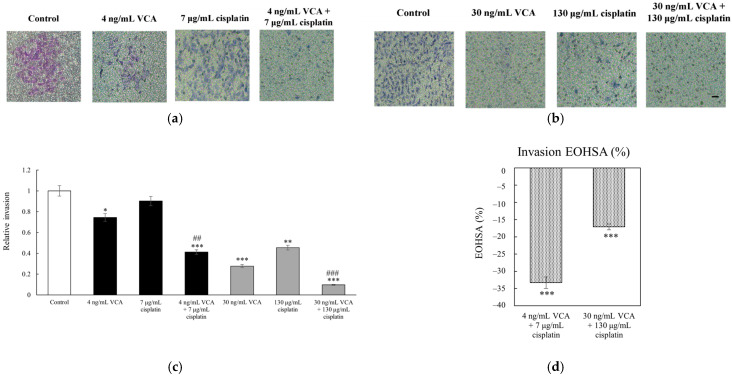
Effects of VCA and cisplatin on MDA-MB-231 cell invasion. Cells were treated with (**a**) 4 ng/mL VCA or 7 μg/mL cisplatin or 4 ng/mL VCA plus 7 μg/mL cisplatin and (**b**) 30 ng/mL VCA or 130 μg/mL cisplatin or 30 ng/mL VCA plus 130 μg/mL cisplatin for 12 h. Invaded cells were observed using a light microscope (magnification, ×100; scale bar, 100 μm). (**c**) The number of invaded cells was quantified using ImageJ software. Data are presented as mean ± SD from three independent experiments. The number of invaded cells in the control group was set as 1. (**d**) Excess Over Highest Single Agent (EOHSA) analysis of combination treatments. EOHSA values below 0 indicate synergistic effects. Data are presented as mean ± SD with 95% confidence intervals from three independent experiments. Statistical significance was determined using one-way ANOVA followed by Tukey’s post hoc test. * *p* < 0.05, ** *p* < 0.01, *** *p* < 0.001 compared to control; ## *p* < 0.01, ### *p* < 0.001 compared to respective single treatments at the same concentrations.

We evaluated the effects of VCA and cisplatin on MDA-MB-231 cell metastatic potential using Transwell migration and invasion assays. In these assays, cells were seeded in the upper chamber and allowed to migrate through membrane pores (migration) or a Matrigel barrier (invasion) towards a chemoattractant in the lower chamber, providing quantitative measures of cell motility and invasive capacity. For migration, cells were seeded in Transwell inserts and treated with VCA (4 or 30 ng/mL), cisplatin (7 or 130 μg/mL), or their combinations for 12 h. All experiments were performed in triplicate, with data presented as means ± 95% confidence intervals. As shown in Figure 4, the untreated cells exhibited high migratory capacity. Low-dose VCA (4 ng/mL) or cisplatin (7 μg/mL) alone did not significantly affect migration. However, their combination reduced migration by 50.42% (*p* < 0.01 vs. control, *p* < 0.05 vs. single agents). Higher doses were more effective: 30 ng/mL VCA decreased migration by 67.05% (*p* < 0.001), and 130 μg/mL cisplatin by 57.14% (*p* < 0.001). The high-dose combination reduced migration by 87.93% (*p* < 0.001 vs. control, *p* < 0.01 vs. single agents). Additionally, in the invasion assay, untreated cells showed strong invasive capacity (Figure 5). Low-dose VCA (4 ng/mL) reduced invasion by 27.71% (*p* < 0.05), while low-dose cisplatin had no significant effect. Their combination suppressed invasion by 61.44% (*p* < 0.001 vs. control, *p* < 0.01 vs. single agents). Higher doses were more potent: 30 ng/mL VCA reduced invasion by 74.05% (*p* < 0.001), and 130 μg/mL cisplatin by 57.84% (*p* < 0.01). The high-dose combination decreased invasion by 90.80% (*p* < 0.001 vs. control and single agents).

### 2.5. Wound Healing Assay in 2D Cells

**Figure 6:**
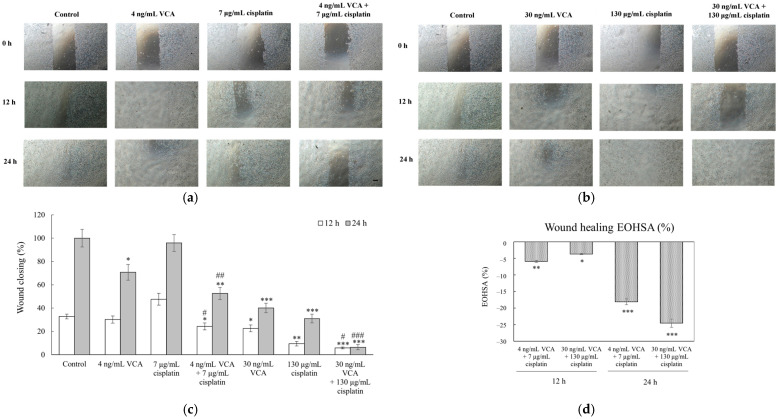
Effects of VCA and cisplatin on MDA-MB-231 cell migration in a wound healing assay. Cells were treated with (**a**) 4 ng/mL VCA, 7 μg/mL cisplatin, or their combinations or (**b**) 30 ng/mL VCA, 130 μg/mL cisplatin, or their combinations for 24 h. Wound closure was measured at 0, 12, and 24 h. Cells were observed using a light microscope (magnification, ×100; scale bar, 100 μm). (**c**) A quantitative bar graph of the closed wound area was generated using image analysis software (ImageJ). (**d**) EOHSA analysis of combination treatments at 12 h and 24 h. EOHSA values below 0 indicate synergistic effects. Data are presented as mean ± SD with 95% confidence intervals from three independent experiments. Statistical significance was determined using one-way ANOVA followed by Tukey’s post hoc test. * *p* < 0.05, ** *p* < 0.01, *** *p* < 0.001 compared to control; # *p* < 0.05, ## *p* < 0.01, ### *p* < 0.001 compared to respective single treatments at the same concentrations.

Using the wound healing assay, which measures the ability of cells to migrate and close an artificial gap in the cell monolayer, we analyzed data from three independent experiments with 95% confidence intervals. Control cells closed 32.78 ± 3.5% of the wound at 12 h and 100 ± 7.5% by 24 h (Figure 6). At 12 h, low doses of VCA (4 ng/mL) or cisplatin (7 μg/mL) alone had no significant effect. However, their combination reduced closure to 24.24 ± 3.8% (*p* < 0.05 vs. control and single agents). Higher doses were more effective: 30 ng/mL VCA reduced closure to 22.58 ± 2.9% (*p* < 0.05), and 130 μg/mL cisplatin to 9.44 ± 2.5% (*p* < 0.01). The high-dose combination was most potent, with only 5.76 ± 1.8% closure (*p* < 0.001 vs. control, *p* < 0.01 vs. single agents). At 24 h, 4 ng/mL VCA reduced closure to 70.71 ± 6.8% (*p* < 0.05), while 7 μg/mL cisplatin had no significant effect. Their combination decreased closure to 52.59 ± 5.3% (*p* < 0.01 vs. control and single agents). When the cells were treated with higher doses, 30 ng/mL VCA and 130 μg/mL cisplatin alone reduced closure to 40.12 ± 4.1% and 30.99 ± 3.7%, respectively (both *p* < 0.001). The high-dose combination nearly stopped migration, with 6.43 ± 2.2% closure (*p* < 0.001 vs. control and single agents).

### 2.6. Synergy Analysis of 2D Cells

To quantify the synergistic interactions of VCA and cisplatin on cell migration, invasion, and wound healing, we performed Excess Over Highest Single Agent (EOHSA) analysis. In the migration assay (Figure 4d), the low-dose combination (4 ng/mL VCA + 7 μg/mL cisplatin) showed an EOHSA of −19.77%, while the high-dose combination (30 ng/mL VCA + 130 μg/mL cisplatin) exhibited an EOHSA of −20.88%. For the invasion assay (Figure 5d), the low-dose combination demonstrated an EOHSA of −33.33%, and the high-dose combination showed an EOHSA of −17.11%. In the wound healing assay at 24 h (Figure 6d), the low-dose combination had an EOHSA of −23.32%, while the high-dose combination showed an EOHSA of −3.68%. These negative EOHSA values across all assays indicate synergistic interactions between VCA and cisplatin in inhibiting cell migration, invasion, and wound healing.

### 2.7. Expression of Apoptosis-Related Proteins via Western Blotting in 2D Cells

**Figure 7:**
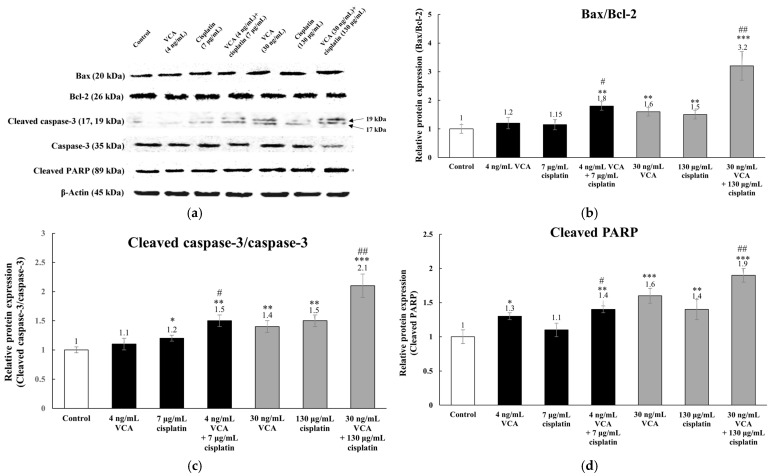
Effects of VCA and cisplatin on apoptosis-related protein expression in MDA-MB231 cells. (**a**) Representative Western blot images showing protein expression levels of apoptosis-related proteins (Bcl-2, Bax, cleaved caspase-3, caspase-3, and cleaved PARP). Quantification of relative protein expression levels of (**b**) Bax/Bcl-2 ratio, (**c**) cleaved caspase-3/caspase-3 ratio, and (**d**) cleaved PARP. Protein expression was normalized to β-actin, which served as a loading control. Cleaved caspase-3 is shown as two distinct bands at 19 kDa and 17 kDa, representing different cleavage products, while full-length caspase-3 is detected at 35 kDa. Data are presented as mean ± SD with 95% confidence intervals from three independent experiments. Statistical significance was determined using one-way ANOVA followed by Tukey’s post hoc test. * *p* < 0.05, ** *p* < 0.01, *** *p* < 0.001 compared to control; # *p* < 0.05, ## *p* < 0.01 compared to respective single treatments at the same concentrations.

For apoptotic markers (Figure 7), untreated MDA-MB-231 cells showed low levels of pro-apoptotic markers Bax, cleaved caspase-3, and cleaved PARP, with high levels of the anti-apoptotic marker Bcl-2 and inactive caspase-3. This profile aligns with their inherent chemoresistance. The combination treatments significantly altered the expression of these markers. The Bax/Bcl-2 ratio increased by 1.83 ± 0.32-fold with the low-dose combination (*p* < 0.01) and 3.24 ± 0.47-fold with the high-dose combination (*p* < 0.001). Similar significant increases were observed in the ratios of cleaved caspase-3 to caspase-3 (1.52 ± 0.23-fold for low-dose, *p* < 0.01; 2.13 ± 0.28-fold for high-dose, *p* < 0.001) and in cleaved PARP expression (1.43 ± 0.19-fold for low-dose, *p* < 0.01; 1.94 ± 0.31-fold for high-dose, *p* < 0.001).

### 2.8. Expression of Metastasis-Related Proteins via Western Blotting in 2D Cells

**Figure 8:**
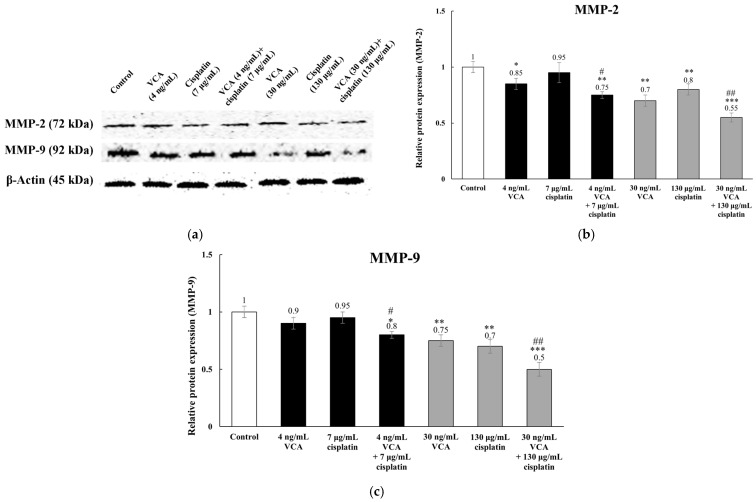
(**a**) Representative Western blot images showing protein expression of metastasis-related proteins MMP-2 and MMP-9. Densitometric analysis of (**b**) MMP-2 and (**c**) MMP-9 protein levels normalized to β-actin. Data are presented as mean ± SD with 95% confidence intervals from three independent experiments. Statistical significance was determined using one-way ANOVA followed by Tukey’s post hoc test. * *p* < 0.05, ** *p* < 0.01, *** *p* < 0.001 compared to control; # *p* < 0.05, ## *p* < 0.01 compared to respective single treatments at the same concentrations.

The combined effects of VCA and cisplatin on MDA-MB-231 cell metastatic potential were analyzed by assessing MMP-2 and MMP-9 expression using Western blot. These matrix metalloproteinases are key enzymes involved in breaking down the extracellular matrix during cancer cell invasion. Their expression levels were normalized to β-actin and quantified using densitometry analysis. Untreated cells exhibited high basal levels of both MMP-2 and MMP-9, consistent with their invasive phenotype (Figure 8). VCA treatment (4 ng/mL, 48 h) decreased MMP-2 expression by 16.3% ± 7.8% (*p* < 0.05) but did not significantly affect MMP-9. Cisplatin (7 μg/mL) did not significantly alter either protein’s expression. Combined treatment (4 ng/mL VCA + 7 μg/mL cisplatin, 48 h) reduced MMP-2 by 26.7% ± 6.9% (*p* < 0.01) and MMP-9 by 21.4% ± 7.6% (*p* < 0.05) compared to the controls. Higher concentrations showed greater effects. VCA (30 ng/mL) alone decreased MMP-2 by 31.2% ± 5.8% and MMP-9 by 24.6% ± 6.9% (*p* < 0.01). Cisplatin (130 μg/mL) reduced MMP-2 by 19.8% ± 7.5% and MMP-9 by 29.3% ± 5.7% (*p* < 0.01). The high-dose combination (30 ng/mL VCA + 130 μg/mL cisplatin) diminished MMP-2 by 46.3% ± 4.8% and MMP-9 by 51.2% ± 5.9% (*p* < 0.001), significantly more than either agent alone (*p* < 0.01). These findings suggest that the VCA and cisplatin combination more effectively suppresses metastasis-related protein expression in MDA-MB-231 cells than individual treatments.

### 2.9. Expression of EMT-Related Proteins via Western Blotting in 2D Cells

**Figure 9:**
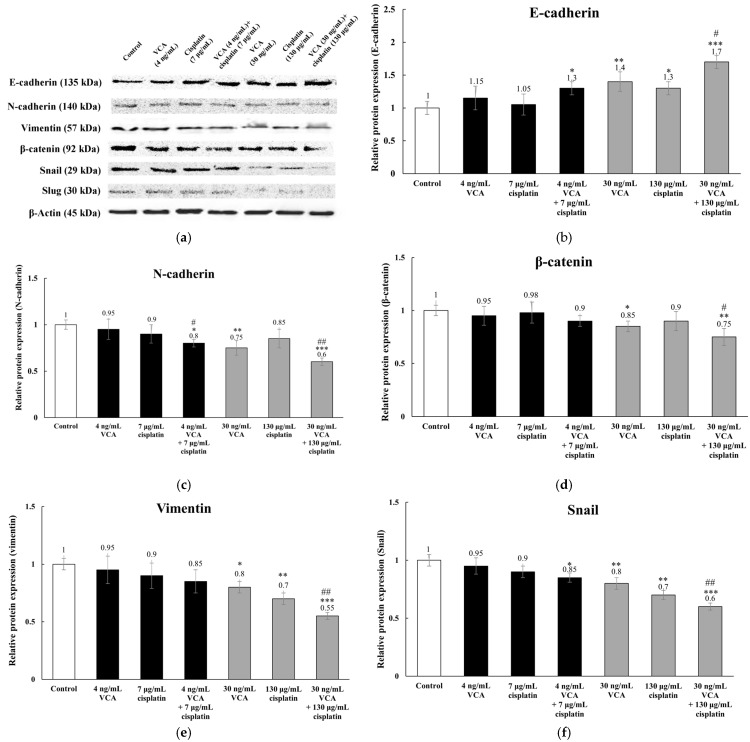

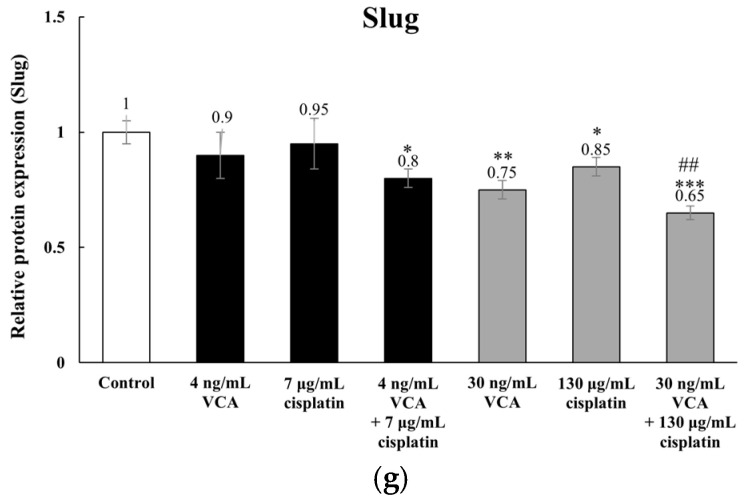
Effects of VCA and cisplatin on EMT marker expressions in MDA-MB231 cells. (**a**) Representative Western blot images showing protein expression of EMT markers. Densitometric analysis of relative protein expression levels of (**b**) E-cadherin, (**c**) N-cadherin, (**d**) β-catenin, (**e**) vimentin, (**f**) Snail, and (**g**) Slug. All protein expressions were normalized to β-actin, which served as an internal loading control. Data are presented as mean ± SD with 95% confidence intervals from three independent experiments. Statistical significance was determined using one-way ANOVA followed by Tukey’s post hoc test. * *p* < 0.05, ** *p* < 0.01, *** *p* < 0.001 compared to control; # *p* < 0.05, ## *p* < 0.01 compared to respective single treatments at the same concentrations.

We investigated VCA and cisplatin effects on EMT markers in MDA-MB-231 cells via Western blot analysis. We examined epithelial marker E-cadherin, mesenchymal markers N-cadherin and vimentin, and EMT transcription factors β-catenin, Snail, and Slug. Alterations in these proteins reflect transitions between epithelial and mesenchymal phenotypes, influencing cell motility and invasive capacity. Untreated cells exhibited high N-cadherin and vimentin, with low E-cadherin, consistent with their mesenchymal phenotype (Figure 9). Combination treatments elicited the most pronounced effects on EMT markers. The low-dose combination (4 ng/mL VCA + 7 μg/mL cisplatin) increased E-cadherin by 28.9% ± 19.4% and decreased N-cadherin by 19.3% ± 8.7% (both *p* < 0.05), while individual low-dose treatments showed no significant effects. High-dose treatments showed more significant effects. VCA (30 ng/mL) increased E-cadherin by 38.6% ± 21.3% (*p* < 0.01) and decreased N-cadherin and vimentin (*p* < 0.01 and *p* < 0.05, respectively). Cisplatin (130 μg/mL) increased E-cadherin and decreased vimentin (both *p* < 0.05). The high-dose combination (30 ng/mL VCA + 130 μg/mL cisplatin) increased E-cadherin by 68.3% ± 24.1% and decreased N-cadherin by 39.2% ± 6.8% and vimentin by 43.7% ± 5.9%. Regarding EMT-regulating transcription factors, low doses had no significant effect. VCA (30 ng/mL) decreased β-catenin, Snail, and Slug by 14.3% ± 7.5% (*p* < 0.05), 24.1% ± 7.6% (*p* < 0.01), and 18.9% ± 7.7% (*p* < 0.05), respectively. Cisplatin (130 μg/mL) reduced Snail by 14.6% ± 8.7% (*p* < 0.05) and Slug by 28.9% ± 6.9% (*p* < 0.01). The high-dose combination decreased β-catenin, Snail, and Slug by 23.8% ± 7.6% (*p* < 0.01), 33.9% ± 6.8% (*p* < 0.001), and 38.7% ± 5.7% (*p* < 0.001), respectively. This reduction was statistically significant compared to the single-agent treatments (*p* < 0.001).

### 2.10. Apoptosis-Related Genes via Real-Time PCR in 2D Cells

**Figure 10:**
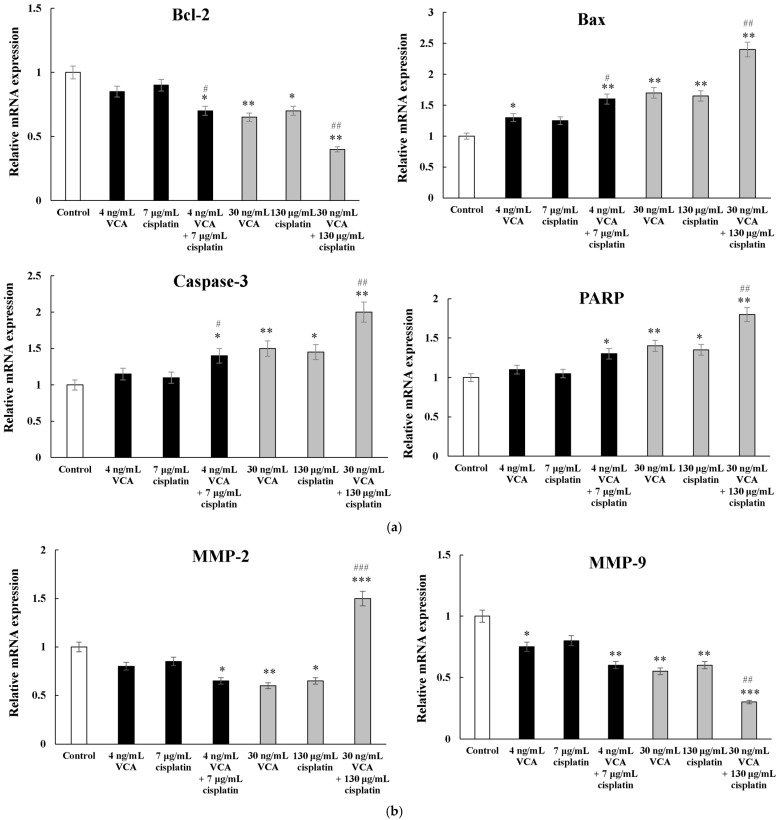

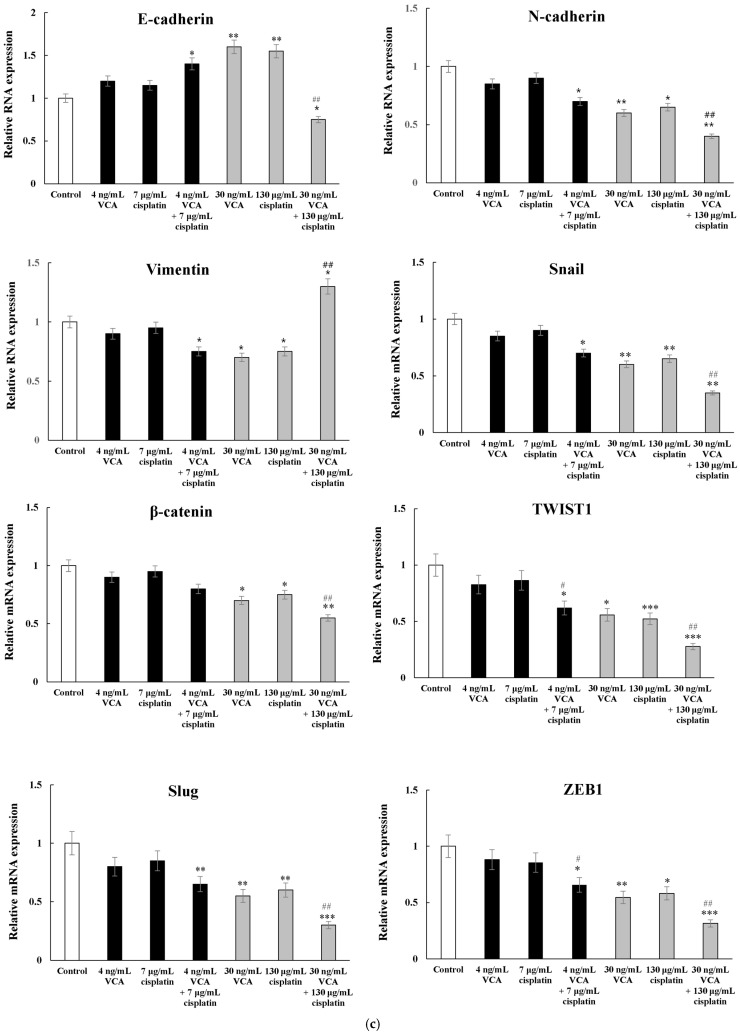

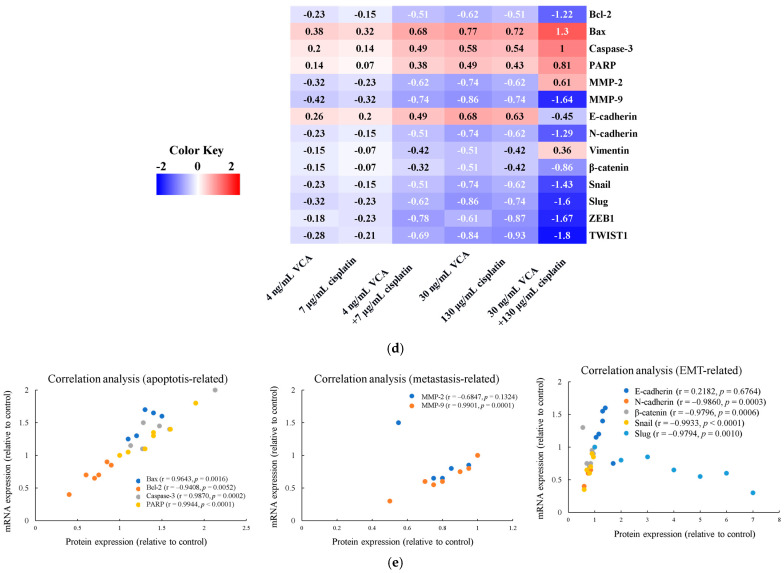
Effects of VCA and cisplatin on gene expression in MDA-MB-231 cells. (**a**–**c**) Cells were treated with VCA (IC20 and IC50), cisplatin (IC20 and IC50), or their combinations for 48 h. Gene expression was analyzed by real-time PCR for (**a**) apoptosis-related genes (Bcl-2, Bax, caspase-3, PARP), (**b**) metastasis-related genes (MMP-2, MMP-9), and (**c**) EMT-related genes (E-cadherin, N-cadherin, vimentin, β-catenin, Snail, Slug, ZEB1, Twist). Data are presented as mean ± SD with 95% confidence intervals from three independent experiments. Statistical significance was determined using one-way ANOVA followed by Tukey’s post hoc test. * *p* < 0.05, ** *p* < 0.01, *** *p* < 0.001 compared to control; # *p* < 0.05, ## *p* < 0.01, ### *p* < 0.001 compared to respective single treatments. (**d**) Heatmap representation of gene expression changes across all treatment conditions. The color scale represents log2-fold changes relative to the control, with red indicating upregulation and blue indicating downregulation. (**e**) Correlation analysis between protein and mRNA expression levels in MDA-MB-231 cells. Linear regression analysis revealed strong positive correlations for pro-apoptotic genes (Bax: r = 0.9643, *p* = 0.0016; caspase-3: r = 0.9870, *p* = 0.0002; PARP: r = 0.9944, *p* < 0.0001) and MMP-9 (r = 0.9901, *p* = 0.0001), while significant negative correlations were found for EMT-related genes (N-cadherin: r = −0.9860, *p* = 0.0003; β-catenin: r = −0.9796, *p* = 0.0006; Snail: r = −0.9933, *p* < 0.0001; Slug: r = −0.9741, *p* = 0.0010).

VCA and cisplatin treatments altered apoptosis-related gene expression (Figure 10a). Using real-time PCR analysis, we measured the expression of the anti-apoptotic gene Bcl-2 and pro-apoptotic genes Bax, caspase-3, and PARP. Expression levels were normalized to GAPDH, used as an internal control, and results are reported as fold changes relative to untreated controls. Bcl-2 decreased in all groups, with the most significant reduction observed in the high-dose combination (30 ng/mL VCA + 130 μg/mL cisplatin, 0.43 ± 0.06-fold, *p* < 0.01). Pro-apoptotic genes Bax and caspase-3 were upregulated, particularly with combination treatments. The high-dose combination significantly increased Bax (2.47 ± 0.22-fold, *p* < 0.01) and caspase-3 (1.98 ± 0.15-fold, *p* < 0.01) compared to the control. PARP expression also increased with combination treatments, reaching 1.76 ± 0.13-fold (*p* < 0.01) with the high-dose combination. These results indicate that VCA and cisplatin, particularly when combined, alter apoptosis-related gene expression towards a pro-apoptotic state in MDA-MB-231 cells.

### 2.11. Metastasis-Related Genes via Real-Time PCR in 2D Cells

The treatments differentially affected metastasis-related genes MMP-2 and MMP-9 (Figure 10b). MMP-9 expression significantly decreased in all treatment groups, with the high-dose combination causing the largest reduction (0.32 ± 0.04-fold, *p* < 0.01). MMP-2 expression showed a more complex pattern. It decreased in single treatments and the low-dose combination (0.78 ± 0.09-fold, *p* < 0.05). However, the high-dose combination treatment statistically significantly increased MMP-2 expression (1.53 ± 0.17-fold, *p* < 0.01). These results suggest that while VCA and cisplatin generally reduce metastasis-related gene expression, high-dose combination treatment may have divergent effects on specific genes.

### 2.12. EMT-Related Genes via Real-Time PCR in 2D Cells

As shown in Figure 10c, E-cadherin expression increased in most groups (1.32 ± 0.11-fold, *p* < 0.05 for the low-dose combination) but decreased with the high-dose combination (0.73 ± 0.08-fold, *p* < 0.05). N-cadherin decreased in all treatments, with the most significant reduction in the high-dose combination (0.41 ± 0.05-fold, *p* < 0.01). Vimentin decreased in most treatments (0.68 ± 0.07-fold, *p* < 0.05 for the low-dose combination) but increased significantly in the high-dose combination (1.28 ± 0.14-fold, *p* < 0.05). Snail and Slug expression consistently decreased, with the high-dose combination showing the most significant reductions (Snail: 0.37 ± 0.04-fold, Slug: 0.33 ± 0.03-fold, both *p* < 0.01). Additionally, real-time PCR analysis revealed that treatment with VCA and cisplatin, both alone and in combination, significantly affected the expression of TWIST1 and ZEB1. Treatment with 30 ng/mL VCA alone reduced TWIST1 expression to 0.56 ± 0.06-fold (*p* < 0.05) and ZEB1 to 0.58 ± 0.06-fold (*p* < 0.05) compared to the controls. Cisplatin (130 μg/mL) showed similar effects, decreasing TWIST1 to 0.55 ± 0.06-fold (*p* < 0.001) and ZEB1 to 0.52 ± 0.06-fold (*p* < 0.05). The combination of VCA (30 ng/mL) and cisplatin (130 μg/mL) exhibited a statistically significant effect, downregulating TWIST1 to 0.32 ± 0.06-fold (*p* < 0.001) and ZEB1 to 0.32 ± 0.04-fold (*p* < 0.001) compared to the control, which was statistically significantly lower than either agent alone (*p* < 0.01 for both genes).

### 2.13. Heatmap Analysis of 2D Cells

Heatmap analysis (Figure 10d) provides a detailed visualization of gene expression changes in response to VCA and cisplatin treatments. This analysis reveals distinct clusters of genes with coordinated expression patterns, providing insights into the molecular mechanisms underlying the effects of these treatments. Notably, pro-apoptotic genes (Bax, caspase-3, PARP) consistently showed upregulation across treatments, with the most pronounced effects observed in the high-dose combination group. In contrast, the anti-apoptotic gene Bcl-2 exhibited consistent downregulation. This pattern supports the enhanced pro-apoptotic effects of the combination therapy observed in our cellular assays. Metastasis-related genes (MMP-2, MMP-9) and most EMT-related genes (N-cadherin, β-catenin, Snail, Slug, ZEB1, TWIST1) displayed a general trend of downregulation, particularly in the high-dose combination treatment. This aligns with our observations of reduced cell migration and invasion in functional assays. Interestingly, we observed distinct patterns between mRNA and protein expression levels of EMT markers in the high-dose combination treatment. While Western blot analysis showed an increase in E-cadherin protein levels and a decrease in vimentin protein levels, PCR data revealed opposite trends. This discrepancy between transcriptional and translational levels indicates complex post-transcriptional regulation of EMT markers during high-dose combination treatment. Despite these differences, both protein and mRNA levels of EMT transcription factors (β-catenin, Snail, Slug, ZEB1, and TWIST1) showed consistent downregulation, indicating that the combination treatment effectively suppresses the core EMT regulatory machinery.

### 2.14. Correlation Analysis Between Protein and mRNA Expression Levels in 2D Cells

To gain insights into the relationship between protein and mRNA expression levels and potential post-transcriptional regulation, we performed a correlation analysis for all investigated proteins (Figure 10e). Significant positive correlations were identified for Bcl-2, Bax, caspase-3, PARP, and MMP-9 (r > 0.94, *p* < 0.01), suggesting that their protein expression is predominantly governed by transcriptional regulation. In contrast, significant negative correlations were found for N-cadherin, β-catenin, Snail, and Slug (r < −0.94, *p* < 0.01), suggesting the presence of post-transcriptional regulatory mechanisms. Vimentin exhibited a moderately strong negative correlation (r = −0.8660, *p* = 0.0255), whereas MMP-2 and E-cadherin showed no statistically significant correlations (*p* > 0.05).

### 2.15. Effect of VCA and Cisplatin on Angiogenesis-Related Factors in 2D Cells

**Figure 11:**
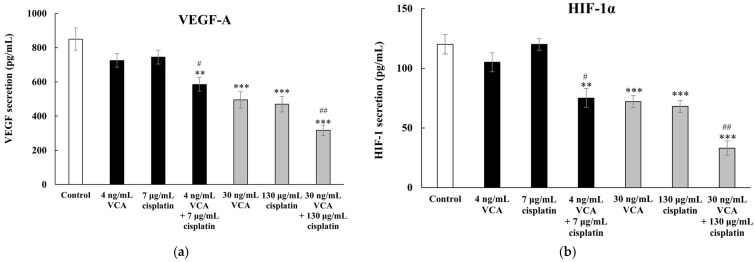
Effects of VCA and cisplatin on angiogenesis-related factors. Secreted levels of (**a**) VEGF-A and (**b**) HIF-1α measured by ELISA. Data are presented as mean ± SD with 95% confidence intervals from three independent experiments. Statistical significance was determined using one-way ANOVA followed by Tukey’s post hoc test. ** *p* < 0.01, *** *p* < 0.001 compared to control; # *p* < 0.05, ## *p* < 0.01 compared to respective single treatments.

ELISA analysis from three independent experiments with 95% confidence intervals revealed significant changes in the secretion of angiogenesis-related factors VEGF-A and HIF-1α following treatment with VCA and cisplatin, both alone and in combination (Figure 11a,b). These factors were measured in cell culture supernatants using specific antibody-based detection, with VEGF-A being a key promoter of blood vessel formation and HIF-1α serving as a master regulator of the cellular response to hypoxia. Protein concentrations were quantified using standard curves derived from known amounts of recombinant proteins. In the untreated control cells, the baseline secretion of VEGF-A was 850 ± 75 pg/mL. Treatment with 4 ng/mL VCA resulted in a decrease to 725 ± 65 pg/mL (*p* > 0.05), while 7 μg/mL cisplatin showed a similar effect, reducing VEGF-A levels to 745 ± 68 pg/mL (*p* > 0.05). The combination of 4 ng/mL VCA and 7 μg/mL cisplatin significantly decreased VEGF-A secretion to 585 ± 55 pg/mL (*p* < 0.01 vs. control, *p* < 0.05 vs. single treatments). At higher concentrations, the effects were more significant. VCA at 30 ng/mL significantly reduced VEGF-A secretion to 495 ± 48 pg/mL (*p* < 0.001), while 130 μg/mL cisplatin decreased levels to 470 ± 45 pg/mL (*p* < 0.001). The combination of 30 ng/mL VCA and 130 μg/mL cisplatin exhibited the strongest effect, reducing VEGF-A secretion to 317 ± 40 pg/mL (*p* < 0.001 compared to control, *p* < 0.01 compared to single treatments). HIF-1α secretion followed a similar response pattern to the treatments. Control cells secreted 120 ± 12 pg/mL of HIF-1α. Low-dose treatments with 4 ng/mL VCA and 7 μg/mL cisplatin resulted in modest reductions to 105 ± 10 pg/mL (*p* > 0.05) and 105 ± 11 pg/mL (*p* > 0.05), respectively. Their combination reduced HIF-1α levels to 75 ± 9 pg/mL (*p* < 0.01 compared to control; *p* < 0.05 compared to single treatments). Higher concentrations elicited more substantial effects on HIF-1α secretion. VCA at 30 ng/mL decreased HIF-1α to 72 ± 8 pg/mL (*p* < 0.001), while 130 μg/mL cisplatin reduced levels to 68 ± 7 pg/mL (*p* < 0.001). The combination of 30 ng/mL VCA and 130 μg/mL cisplatin elicited the most substantial reduction in HIF-1α secretion, decreasing levels to 33 ± 6 pg/mL (*p* < 0.001 vs. control, *p* < 0.01 vs. single treatments).

### 2.16. PCA of 2D Culture Data

**Figure 12:**
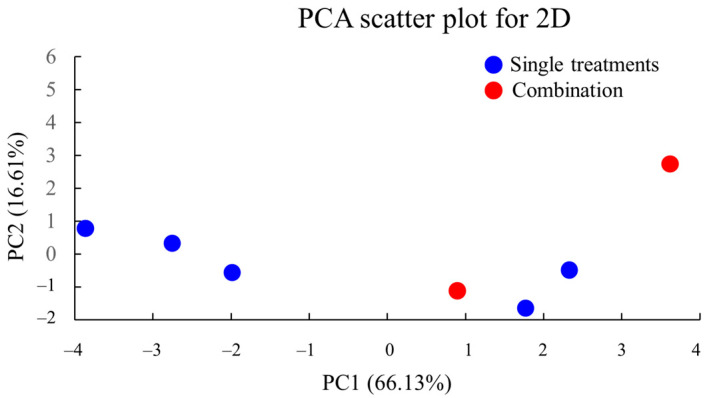
Principal Component Analysis (PCA) of treatment effects on MDA-MB-231 cells. The plot shows the distribution of different treatments along the first two principal components (PC1 and PC2). Single treatments are shown in blue, while combination treatments are in red. The position of each point reflects the overall effect of the treatment, with points closer together indicating similar effects. PC1 and PC2 together explain 82.74% of the total variance in the data.

To visualize the overall effects of VCA and cisplatin treatments on MDA-MB-231 cells, PCA was conducted using multiple parameters, including cell viability, apoptosis rates, cell cycle distribution, migration rates, and gene expression levels (Figure 12). PCA is a statistical method that reduces the dimensionality of complex datasets while retaining key patterns in the data. The first two principal components (PC1 and PC2) were selected as they explained the majority of the variance (82.74%), with the distance between points in the PCA plot representing the degree of similarity between treatment conditions. The PCA plot showed distinct clustering of treatment conditions, with the control group positioned separately from the treated groups. The combination treatments, particularly the high-dose combination (30 ng/mL VCA + 130 μg/mL cisplatin), showed the greatest distance from the control, indicating the most pronounced overall effect. The first principal component (PC1) appears to correlate strongly with treatment intensity, while the second principal component (PC2) may represent the differential effects between VCA and cisplatin.

### 2.17. Morphological Analysis of 3D Spheroids

**Figure 13:**
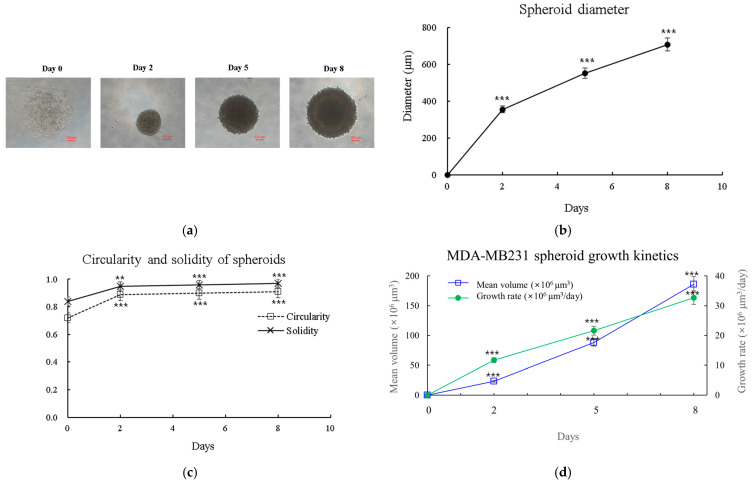
Morphological analysis of MDA-MB-231 3D spheroids. (**a**) Brightfield images of spheroids from day 1 to day 8. Scale bar: 100 μm. (**b**) Plot of spheroid diameter from day 0 to 8. (**c**) Circularity and solidity of spheroids measured from day 0 to day 8. Both circularity and solidity are dimensionless ratios ranging from 0 to 1. (**d**) Growth kinetics of MDA-MB-231 spheroids. The blue line represents the mean spheroid volume over time, fitted to a Gompertz growth model. The green line shows the mean instantaneous growth rate. Error bars represent standard deviation from three independent experiments. ** *p* < 0.01, *** *p* < 0.001 compared to day 0 (one-way ANOVA with Tukey’s post-hoc test).

To characterize the growth and morphology of MDA-MB-231 spheroids, we monitored their formation over 8 days (Figure 13). Immediately after seeding, the cells began to aggregate, forming loose clusters by day 1. The spheroids grew consistently, reaching mean diameters of 355 ± 15 μm by day 2, 552 ± 23 μm by day 5, and 708 ± 30 μm by day 8 (Figure 13b). We further analyzed spheroid morphology by quantifying circularity and solidity (Figure 13c). Circularity, which indicates how closely the spheroid shape approaches a perfect circle, increased from 0.72 ± 0.05 on day 0 to 0.89 ± 0.03 by day 2 and continued to increase slightly to 0.91 ± 0.02 by day 8. Solidity, reflecting the compactness of the spheroid, showed a similar trend, increasing from 0.84 ± 0.04 on day 0 to 0.95 ± 0.02 by day 2 and reaching 0.97 ± 0.01 by day 8. These data indicate that MDA-MB-231 cells form increasingly circular and compact spheroids over time, with the most significant changes occurring within the first 48 h of culture. The growth kinetics of MDA-MB-231 spheroids were monitored over 8 days (Figure 13d). The spheroid volume increased significantly from 0 to 1.86 ± 0.06 × 10^8^ μm^3^ (mean ± SD) over this period (*p* < 0.001, one-way ANOVA). The growth pattern closely followed a Gompertz model (R^2^ = 0.998), with fitted parameters V0 = 1.2 × 10^6^ μm^3^ (95% CI: 0.9–1.5 × 10^6^), A = 0.76 day^−1^ (95% CI: 0.68–0.84), and α = 0.22 day^−1^ (95% CI: 0.18–0.26). The instantaneous growth rate increased from 11.7 ± 0.5 × 10^6^ μm^3^/day on day 2 to 32.7 ± 1.2 × 10^6^ μm^3^/day on day 8. The estimated doubling time was 2.1 ± 0.2 days.

### 2.18. Three-Dimensional Cell Viability

To investigate the effects of VCA and cisplatin on 3D cultures of MDA-MB-231 cells, we performed dose–response analyses using spheroid models. The 3D spheroid model was systematically developed to evaluate drug effects in a physiologically relevant context. MDA-MB-231 cells were first cultured to form uniform spheroids of approximately 500 μm diameter over 4 days. Drug treatments were then applied in three distinct groups: single-agent VCA, single-agent cisplatin, and combination treatments. For each group, both IC50 and IC20 concentrations were tested to assess dose-dependent effects. The treatment duration was optimized to 72 h based on preliminary studies of spheroid growth kinetics and drug response patterns.

**Figure 14:**
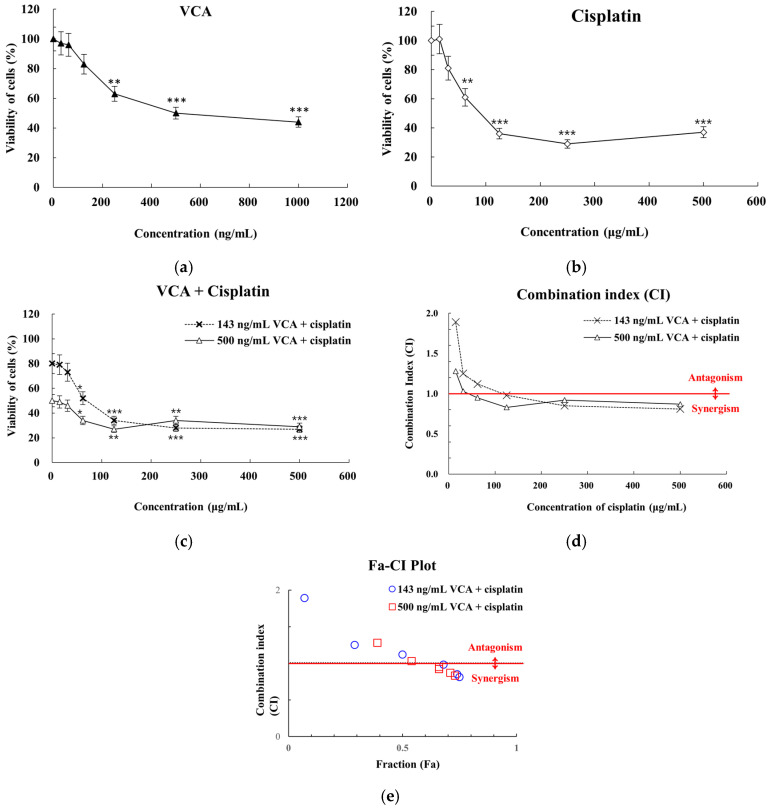
Dose–response curves of VCA and cisplatin in 3D MDA-MB-231 spheroids. Cells were cultured in the absence or presence of different concentrations of (**a**) VCA or (**b**) cisplatin or (**c**) VCA plus cisplatin and the viability was measured by the CellTiter-Glo 3D Cell Viability Assay. (**d**) Combination index (CI) and (**e**) fractional effect (Fa) analysis of VCA and cisplatin combinations. CI < 1 indicates synergy, CI = 1 indicates additive effect, and CI > 1 indicates antagonism. Data are presented as mean ± SD with 95% confidence intervals from three independent experiments. * *p* < 0.05, ** *p* < 0.01, *** *p* < 0.001 compared to the untreated control (one-way ANOVA followed by Dunnett’s post hoc test for single treatments; two-way ANOVA followed by Tukey’s post hoc test for combinations).

Based on initial dose–response studies in 2D cultures, we first established IC50 and IC20 values for both compounds in 3D spheroid models. Cell viability was evaluated using the CellTiter-Glo 3D assay in three independent experiments with 95% confidence intervals. This assay was chosen for its ability to penetrate multilayered spheroids and provide accurate ATP-based viability measurements. As shown in Figure 14a, VCA demonstrated dose-dependent cytotoxicity in 3D cultures, with an IC50 of approximately 500 ng/mL. Cisplatin also showed a dose-dependent effect (Figure 14b), with an IC50 of about 90 μg/mL. For combination studies, we selected both IC50 (500 ng/mL VCA, 90 μg/mL cisplatin) and IC20 (143 ng/mL VCA, 30 μg/mL cisplatin) concentrations to evaluate potential synergistic effects at different inhibitory levels. These IC50 values differed from those observed in 2D cultures (30 ng/mL for VCA and 130 μg/mL for cisplatin), suggesting altered drug responses in the 3D model. Combination treatments of VCA and cisplatin exhibited enhanced cytotoxicity compared to either agent alone (Figure 14c). Two fixed concentrations of VCA (500 ng/mL and 143 ng/mL) were combined with varying concentrations of cisplatin. The combination of 500 ng/mL VCA (IC50) with cisplatin showed greater cytotoxicity than 143 ng/mL VCA (IC20) with cisplatin across all tested concentrations. At the highest cisplatin concentration (500 μg/mL), the combinations with 500 ng/mL and 143 ng/mL VCA reduced cell viability to 29% and 27%, respectively, compared to 37% for cisplatin alone at the same concentration. To quantify the synergistic effects of VCA and cisplatin in 3D cultures, we performed CI analysis (Figure 14d,e). The combinations of 500 ng/mL VCA with various concentrations of cisplatin showed CI values ranging from 0.83 to 1.28. Strong synergism was observed at cisplatin concentrations of 500 μg/mL (CI = 0.87) and 125 μg/mL (CI = 0.83). Nearly additive effects were seen at 250 μg/mL (CI = 0.92) and 62 μg/mL (CI = 0.95), while slight antagonism was observed at lower cisplatin concentrations of 31 μg/mL (CI = 1.03) and 15 μg/mL (CI = 1.28). For the combinations with 143 ng/mL VCA, CI values ranged from 0.81 to 1.89. Strong synergism was observed at higher cisplatin concentrations of 500 μg/mL (CI = 0.81) and 250 μg/mL (CI = 0.85). A nearly additive effect was seen at 125 μg/mL (CI = 0.98), while antagonism was observed at lower cisplatin concentrations of 62 μg/mL (CI = 1.12), 31 μg/mL (CI = 1.25), and 15 μg/mL (CI = 1.89). These findings indicate that the synergistic effects of VCA and cisplatin observed in 2D cultures are partially retained in 3D spheroid models, particularly at higher drug concentrations. However, at lower concentrations, the interaction becomes antagonistic, suggesting a complex, concentration-dependent relationship between the two drugs in 3D culture.

### 2.19. Three-Dimensional Live/Dead Cell Assay

**Figure 15:**
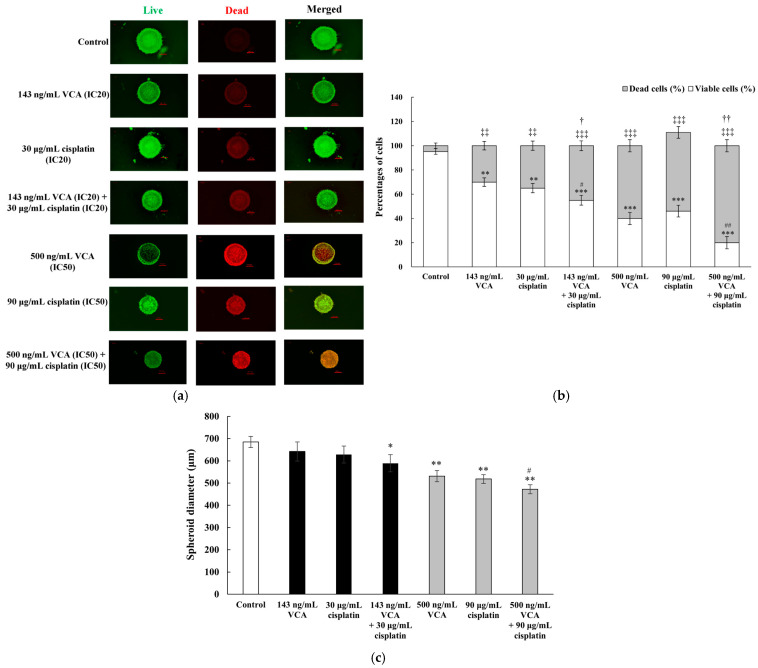
Effects of VCA and cisplatin on 3D MDA-MB-231 spheroids. (**a**) Representative fluorescence microscopy images of live/dead-stained spheroids treated with VCA, cisplatin, or their combinations for 72 h. Green: live cells (calcein AM). Red: dead cells (ethidium homodimer-1). Scale bar: 200 μm. (**b**) Quantification of viable and dead cells based on ImageJ analysis of live/dead staining. Data are presented as mean ± SD from three independent experiments. Statistical significance was determined using one-way ANOVA followed by Tukey’s post hoc test. For viable cells: ** *p* < 0.01, *** *p* < 0.001 compared to control; # *p* < 0.05, ## *p* < 0.01 compared to respective single treatments. For dead cells: ‡‡ *p* < 0.01, ‡‡‡ *p* < 0.001 compared to control; † *p* < 0.05, †† *p* < 0.01 compared to respective single treatments. (**c**) Spheroid diameter measurements after 72 h of treatment. Data in (**b**,**c**) are presented as mean ± SD from three independent experiments. Data are presented as mean ± SD with 95% confidence intervals from three independent experiments. Statistical significance was determined using one-way ANOVA followed by Tukey’s post hoc test. * *p* < 0.05, ** *p* < 0.01 compared to control; # *p* < 0.05 compared to respective single treatments.

Live/dead staining was conducted in three independent experiments with 95% confidence intervals. The results showed a dose-dependent decrease in cell viability across all treatment conditions (Figure 15a). In this assay, spheroids were stained with calcein AM (green fluorescence, indicating live cells) and ethidium homodimer-1 (red fluorescence, indicating dead cells). The spatial distribution of live and dead cells within spheroids provides insights into drug effects, with the periphery typically exhibiting greater sensitivity than the core due to drug penetration gradients. Quantification was performed using ImageJ software to measure the relative intensities of green and red fluorescence. The combination treatments showed enhanced cytotoxicity compared to single-agent treatments, with the high-dose combination (500 ng/mL VCA + 90 μg/mL cisplatin) reducing the proportion of live cells to 42.6 ± 3.7% compared to 61.4 ± 2.9% for VCA alone and 58.9 ± 3.2% for cisplatin alone (*p* < 0.01 for both comparisons). Qualitative assessment of live/dead-stained spheroids showed distinct patterns of cell death distribution (Figure 15b). Control spheroids displayed predominantly green fluorescence, indicating uniformly high viability. IC20 treatments resulted in increased red fluorescence primarily at the spheroid periphery, while IC50 treatments and combinations showed more extensive cell death penetrating into the spheroid core. This pattern highlights differential drug penetration and efficacy between the periphery and core, underscoring the utility of 3D models in evaluating drug effects. Treatment with VCA and cisplatin also led to a reduction in spheroid size (Figure 15c). The high-dose combination treatment resulted in the smallest spheroid diameter (472 ± 35 μm) compared to the control (685 ± 25 μm, *p* < 0.001) and single-agent treatments (VCA: 531 ± 27 μm, cisplatin: 518 ± 30 μm, *p* < 0.01 for both comparisons).

### 2.20. Three-Dimensional Invasion Assay

**Figure 16:**
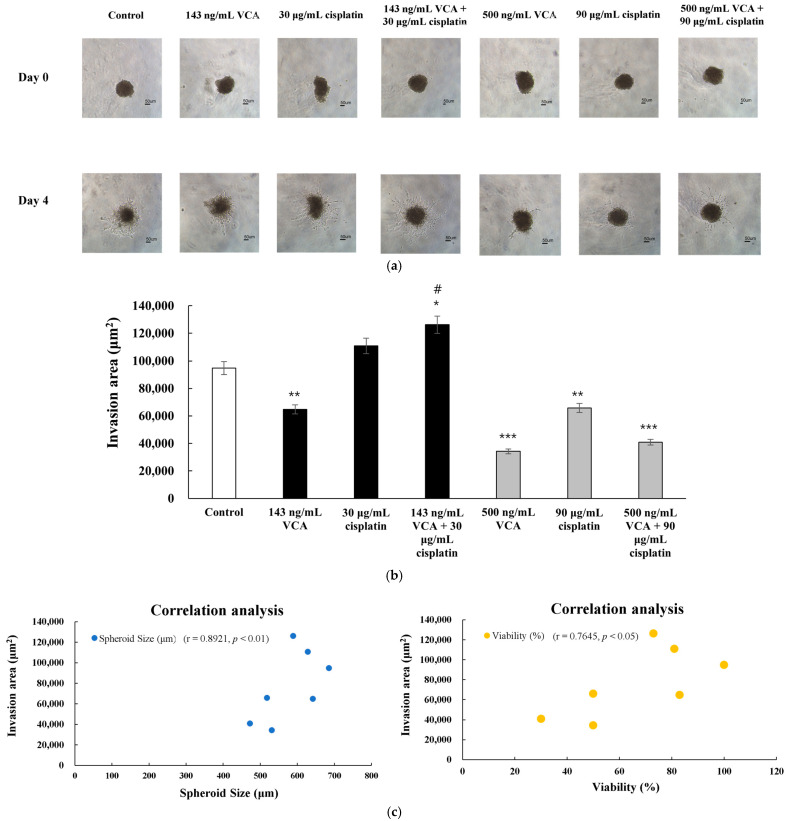
Analysis of 3D MDA-MB231 cell invasion over a 4-day period. (**a**) Representative images of spheroid invasion on day 0 and day 4 for cells treated with 143 ng/mL VCA (IC20), 30 μg/mL cisplatin (IC20), 143 ng/mL VCA plus 30 μg/mL cisplatin, 500 ng/mL VCA (IC50), 90 μg/mL cisplatin (IC50), or 500 ng/mL VCA plus 90 μg/mL cisplatin. Scale bar: 50 μm. (**b**) Quantification of invasion area (difference between day 4 and day 0). (**c**) Correlation analysis of 3D spheroid characteristics in MDA-MB-231 cells. Linear regression analysis showed strong positive correlations between invasion area and spheroid size (r = 0.8921, *p* < 0.01) and between invasion area and cell viability (r = 0.7645, *p* < 0.05). Data are presented as mean ± SD with 95% confidence intervals from three independent experiments. Statistical significance was determined using one-way ANOVA followed by Tukey’s post hoc test. * *p* < 0.05, ** *p* < 0.01, *** *p* < 0.001 compared to control; # *p* < 0.05 compared to respective single treatments at the same concentrations.

The 3D invasion assay was conducted in three independent experiments with 95% confidence intervals. The results demonstrated differential effects of VCA and cisplatin on MDA-MB-231 spheroid invasion (Figure 16). Analysis of spheroid invasion was performed by measuring the area covered by cells migrating out from the central spheroid mass into the surrounding collagen matrix. The invasion area was calculated as the difference between the total area occupied by cells on day 4 and the initial spheroid area on day 0. This measurement specifically evaluates cellular invasion by quantifying both matrix degradation and cell migration through the collagen matrix. Analysis of the invasion area showed complex responses to different treatments (Figure 16b). The combination of 500 ng/mL VCA and 90 μg/mL cisplatin significantly reduced invasion by 56.8% compared to the control (*p* < 0.001) and was more effective than either agent alone (*p* < 0.01 for both comparisons). Interestingly, the low-dose combination (143 ng/mL VCA + 30 μg/mL cisplatin) unexpectedly increased the invasion area by 33.3% compared to the control (*p* < 0.05), suggesting a potential biphasic response. Low-dose VCA (143 ng/mL) reduced invasion by 31.7% (*p* < 0.01), while low-dose cisplatin (30 μg/mL) had no significant effect. High-dose VCA (500 ng/mL) was the most effective single agent, reducing invasion by 63.8% (*p* < 0.001), while high-dose cisplatin (90 μg/mL) reduced invasion by 30.6% (*p* < 0.01). Correlation analysis identified a strong positive relationship between invasion area and spheroid size (r = 0.8921, *p* < 0.01) (Figure 16c), indicating that larger spheroids exhibited greater invasion.

### 2.21. Gene Expression in 3D Spheroids

**Figure 17:**
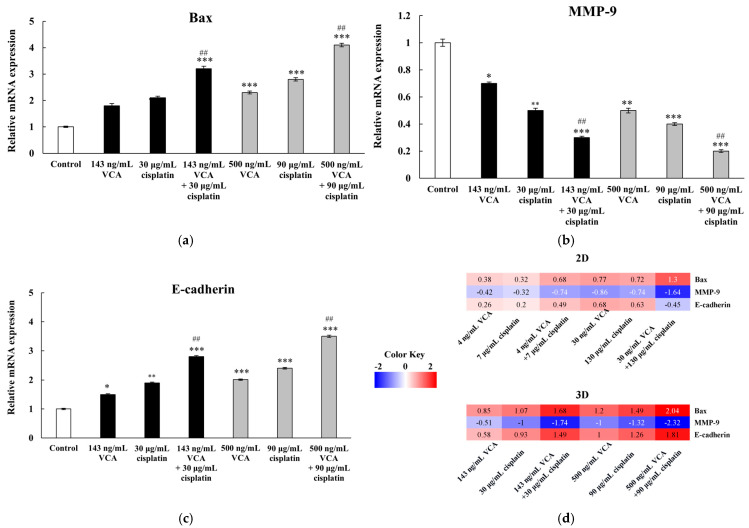
Real-time PCR analysis of (**a**) Bax, (**b**) MMP-9, and (**c**) E-cadherin expression in 3D MDA-MB-231 spheroids. Cells were treated with 143 ng/mL VCA, 30 μg/mL cisplatin, their combination, 500 ng/mL VCA, 90 μg/mL cisplatin, or their combination for 72 h. Gene expression was normalized to GAPDH and expressed as fold change relative to the control. Data are presented as mean ± SD with 95% confidence intervals from three independent experiments. Statistical significance was determined using one-way ANOVA followed by Tukey’s post hoc test. * *p* < 0.05, ** *p* < 0.01, *** *p* < 0.001 compared to control; ## *p* < 0.01 compared to respective single treatments at the same concentrations. (**d**) Heatmap comparison of gene expression changes in 2D and 3D MDA-MB-231 cultures treated with VCA, cisplatin, or their combinations. The color scale represents log2-fold changes relative to the control, with red indicating upregulation and blue indicating downregulation. The intensity of the color corresponds to the magnitude of the change.

To determine whether the effects observed in 2D cultures were preserved in a more physiologically relevant model, we analyzed the expression of key genes in 3D MDA-MB-231 spheroids treated with VCA, cisplatin, or their combinations at low and high concentrations across three independent experiments with 95% confidence intervals (Figure 17). We then compared these results to our previous 2D culture data to highlight culture-dependent effects. RNA was extracted from intact spheroids using specialized protocols designed for 3D cultures to ensure complete lysis and high-quality RNA recovery. Gene expression analysis focused on three representative markers, Bax (apoptosis), MMP-9 (invasion), and E-cadherin (EMT), chosen to reflect different aspects of the cellular response. Expression levels were normalized to GAPDH and compared between 2D and 3D cultures to evaluate culture format-dependent effects.

The pro-apoptotic gene Bax was significantly upregulated with all treatments in both 2D and 3D cultures, though notable differences were observed between the models. In 3D spheroids at low concentrations (143 ng/mL VCA and 30 μg/mL cisplatin), the combination treatment exhibited a higher upregulation (3.2 ± 0.3-fold, *p* < 0.001) compared to the 2D results (2.4 ± 0.2-fold, *p* < 0.001). This trend was consistent at high concentrations (500 ng/mL VCA and 90 μg/mL cisplatin), with 3D spheroids showing a 4.1 ± 0.3-fold increase (*p* < 0.001) compared to 3.2 ± 0.3-fold (*p* < 0.001) in 2D cultures. These results suggest that 3D spheroids may be more sensitive to the pro-apoptotic effects of the combination treatment. The metastasis-related gene MMP-9 showed a significant decrease in expression with all treatments in both culture systems. However, the effect was more significant in 3D spheroids. At low concentrations, the combination treatment in 3D cultures led to a more significant reduction (0.3 ± 0.1-fold, *p* < 0.001) compared to 2D cultures (0.5 ± 0.1-fold, *p* < 0.001). This difference was maintained at high concentrations, with 3D spheroids showing a reduction to 0.2 ± 0.1-fold (*p* < 0.001) compared to 0.3 ± 0.1-fold (*p* < 0.001) in 2D cultures. These findings suggest that the anti-metastatic potential of the combination therapy is more pronounced in 3D models. E-cadherin showed increased expression with all treatments in both 2D and 3D cultures. Interestingly, the upregulation was more significant in 3D spheroids. At low concentrations, the combination treatment resulted in a higher upregulation in 3D cultures (2.8 ± 0.3-fold, *p* < 0.001) compared to 2D cultures (2.1 ± 0.2-fold, *p* < 0.001). This trend persisted at high concentrations, where 3D spheroids exhibited a 3.5 ± 0.3-fold increase (*p* < 0.001) compared to 2.8 ± 0.3-fold (*p* < 0.001) in 2D cultures. These results suggest that the potential reversal of EMT by the combination therapy may be more evident in the 3D model.

### 2.22. PCA of Treatment Effects in 3D Culture 

**Figure 18:**
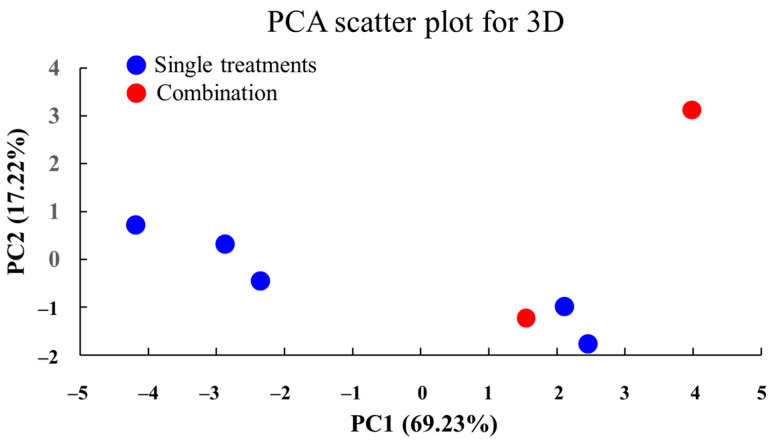
Principal Component Analysis (PCA) of treatment effects on MDA-MB-231 cells in 3D culture. The plot shows the distribution of different treatments along the first two principal components (PC1 and PC2) based on cell viability, live/dead cell data, invasion data, and gene expression data (Bax, MMP-9, and E-cadherin). Single treatments are shown in blue, while combination treatments are in red. The position of each point reflects the overall effect of the treatment, with points closer together indicating similar effects. PC1 and PC2 together explain 86.45% of the total variance in the data.

To visualize the effects of VCA and cisplatin treatments on MDA-MB-231 cells in 3D culture, PCA was conducted using cell viability, live/dead cell, invasion, and gene expression data for Bax, MMP-9, and E-cadherin (Figure 18). This multivariate analysis approach integrates multiple experimental endpoints to reveal overall patterns in the response to treatment. The first two principal components (PC1: 69.23%, PC2: 17.22%) accounted for 86.45% of the total variance, with the PCA plot positioning points based on the overall similarity or differences among treatment conditions in 3D culture. The separation between single and combination treatments along PC1 indicates distinct response patterns, while the distribution along PC2 suggests dose-dependent effects. The first two principal components (PC1 and PC2) accounted for 86.45% of the total variance in the data, with PC1 explaining 69.23% and PC2 explaining 17.22%. The PCA plot reveals a distinct clustering of treatment conditions, with the control group positioned separately from the treated groups. Combination treatments, particularly the high-dose combination (500 ng/mL VCA + 90 μg/mL cisplatin), exhibited the greatest distance from the control, indicating the most pronounced overall effect. The single treatments with higher concentrations (500 ng/mL VCA and 90 μg/mL cisplatin) were positioned between the control and the combination treatments, suggesting a dose-dependent effect. Interestingly, the low-dose combination treatment (143 ng/mL VCA + 30 μg/mL cisplatin) occupied a distinct position in the PCA plot, separate from both the control and single treatments at comparable concentrations. This suggests that even at lower doses, the combination of VCA and cisplatin produces a unique cellular response that is different from either agent alone.

## 3. Discussion

This study investigated the potential synergistic effects of VCA, a compound extracted from mistletoe, and cisplatin on MDA-MB-231 cells, a highly aggressive and metastatic TNBC cell line. Our findings demonstrate a potent, dose-dependent synergistic effect of the VCA–cisplatin combination on TNBC cell viability. This synergy likely arises from the complementary mechanisms of action: cisplatin’s induction of DNA damage and subsequent cell cycle arrest [47] combined with VCA’s multifaceted effects on protein synthesis, oxidative stress, and cellular signaling pathways [69,77]. Importantly, our comparative analysis using MCF-10A normal breast epithelial cells revealed that both compounds demonstrate selective cytotoxicity towards cancer cells. The substantially higher IC50 values observed in MCF-10A cells (8.3-fold higher for VCA and 1.4-fold higher for cisplatin) suggest a favorable therapeutic window for this combination treatment. This selectivity might be attributed to the different metabolic states and stress response mechanisms between normal and cancer cells, as well as VCA’s reported ability to specifically target transformed cells through differential glycosylation patterns [78,79,80].

The cytotoxic effects were confirmed by both MTT and crystal violet assays, with the latter providing a direct measure of cell numbers. The synergistic effect was consistently observed across these assays at clinically relevant concentrations. The combination also showed significant synergistic inhibition of cell migration, invasion, and wound healing in MDA-MB-231 cells. EOHSA analysis revealed strong synergy, particularly in the invasion assay for the low-dose combination (EOHSA −33.33%). Notably, this anti-metastatic efficacy was achieved at lower concentrations that could minimize cisplatin-associated toxicities, a crucial advantage for TNBC management [81].

The VCA–cisplatin combination demonstrates a marked enhancement in apoptosis induction through multiple pathways. VCA modulates pro- and anti-apoptotic protein expression, particularly the Bcl-2 family and caspases [62,82,83], while cisplatin triggers p53-mediated apoptotic responses through DNA damage [84]. The high-dose combination (30 ng/mL VCA and 130 μg/mL cisplatin) significantly increased the Bax/Bcl-2 ratio (3.24-fold), cleaved the caspase-3/caspase-3 ratio (2.13-fold), and cleaved PARP (1.94-fold) (all *p* < 0.001). These changes indicate a strong shift towards pro-apoptotic signaling, potentially overcoming TNBC’s apoptosis resistance [85]. Additionally, the combination induced G2/M phase arrest through modulation of p21, cyclin B1, and Cdc2 [86], representing a novel finding in TNBC cells. This dual action on apoptosis and cell cycle progression explains the enhanced efficacy of the combination therapy [87].

The anti-metastatic effects of the VCA–cisplatin combination are particularly noteworthy in the context of TNBC, where metastasis remains a primary cause of mortality [81]. These effects correlate with significant changes in metastasis-related proteins. The combination downregulated MMP-2 and MMP-9, key enzymes in extracellular matrix degradation [81,88], explaining its anti-invasive effects. Regarding EMT markers, the treatment increased E-cadherin while decreasing N-cadherin and vimentin levels. It also suppressed EMT-regulating transcription factors (β-catenin, Snail, and Slug) [89]. These molecular changes, particularly the reversal of cadherin switching, provide mechanistic support for the observed reduction in cell migration and invasion.

Our gene expression analysis revealed complex molecular impacts of the VCA–cisplatin combination. In the high-dose combination group, pro-apoptotic genes were significantly upregulated, with Bax and caspase-3 increasing 2.47-fold and 1.98-fold, respectively (*p* < 0.01), aligning with our protein-level data. The significant downregulation of EMT-related genes (N-cadherin, β-catenin, Snail, Slug, ZEB1, and TWIST1) suggests effective inhibition of the EMT process [90]. Furthermore, the reduction in expression and secretion of angiogenesis-related factors VEGF-A and HIF-1α implies that the combination therapy might also inhibit tumor vascularization [91]. This dual action on both EMT and angiogenesis could potentially enhance the overall anti-cancer effect of the treatment [92]. Interestingly, we observed discrepancies between mRNA and protein levels for some genes (e.g., MMP-2, E-cadherin, vimentin) in the high-dose combination group. These differences showed distinct concentration-dependent patterns—at lower doses (4 ng/mL VCA + 7 μg/mL cisplatin), transcriptional and translational changes were largely concordant, while higher doses (30 ng/mL VCA + 130 μg/mL cisplatin) triggered more complex regulatory mechanisms. This concentration-dependent discrepancy aligns with recent findings by Harvey et al. [93], who demonstrated that high-dose therapeutic stress can trigger extensive post-transcriptional modifications affecting protein stability. Furthermore, Perl et al. [94] revealed that such mRNA-protein level discrepancies often involve altered protein degradation pathways specifically activated under high drug concentrations. These differences suggest complex regulatory mechanisms, including post-transcriptional and post-translational processes [95,96]. Such discrepancies highlight the importance of examining both mRNA and protein levels when assessing drug effects.

The heatmap analysis (Figure 10d) revealed coordinated responses in apoptosis, metastasis, and EMT-related pathways across treatments. Our analysis of protein and mRNA levels (Figure 10e) identified two distinct patterns: direct transcriptional regulation (e.g., Bax, caspase-3) and post-transcriptional regulation (e.g., Bcl-2, N-cadherin). The high-dose combination treatment showed notable differences between mRNA and protein levels of EMT markers. These differences likely result from protein stability changes, post-transcriptional modifications, and transcriptional feedback mechanisms—patterns similar to those observed in other cancer cell stress response studies [97,98]. The consistent downregulation of EMT transcription factors at both the mRNA and protein levels indicates that the combination therapy suppresses the core EMT program while affecting structural EMT markers through multiple regulatory mechanisms.

The biomarkers examined in this study hold significant potential for clinical applications beyond their mechanistic implications. The Bax/Bcl-2 ratio has been established as a strong prognostic indicator in breast cancer patients, with higher ratios correlating with better survival outcomes [99]. Our observation of an increased Bax/Bcl-2 ratio under combination treatment suggests its potential utility in monitoring therapeutic response. Similarly, MMP-2 and MMP-9 levels have been shown to correlate with disease progression and metastatic potential in TNBC patients, making them valuable predictive biomarkers [100]. The significant reduction in these MMPs following combination treatment could serve as an early indicator of treatment efficacy. The EMT markers analyzed in our study (E-cadherin, N-cadherin, vimentin) could be particularly useful for patient stratification, as their expression patterns often predict therapy response and progression-free survival in TNBC [101,102]. The modulation of these markers by the VCA–cisplatin combination suggests their potential utility in identifying patients most likely to benefit from this treatment approach [103]. These findings also highlight potential therapeutic strategies targeting EMT pathways in combination with VCA–cisplatin treatment. Furthermore, monitoring these biomarkers during treatment could provide valuable information about therapeutic efficacy and help guide treatment decisions.

Several key limitations affect the interpretation of our gene expression data. The use of three independent experiments with technical triplicates provides initial insights but may have limited statistical power. Our analysis faced three main technical challenges: (1) gene expression measurements at specific time points may miss dynamic transcriptional changes; (2) GAPDH normalization could be affected by experimental conditions; and (3) inherent variability exists between experiments. Additionally, the discrepancies we observed between mRNA and protein levels for genes like MMP-2, E-cadherin, and vimentin in the high-dose combination group highlight the complex relationship between transcript levels and functional outcomes. While our in vitro data reveal significant changes in key regulatory pathways, validation in in vivo models is essential, as the tumor microenvironment may differently influence gene expression patterns. These limitations emphasize the need for future in vivo studies using multiple experimental approaches.

The PCA of both 2D and 3D culture data provides a comprehensive view of the multifaceted effects of VCA and cisplatin on MDA-MB-231 cells. The clear separation of treatment groups in the PCA plots underscores the distinct cellular responses elicited by these compounds, both alone and in combination. Notably, the positioning of combination treatments, particularly the high-dose combination, furthest from the control group corroborates our findings of synergistic effects.

Our 3D spheroid experiments provided valuable insights into the efficacy of VCA and cisplatin in a more physiologically relevant model. The MDA-MB-231 spheroids formed cohesive, well-organized structures with a final size of approximately 700 μm, allowing for the development of physiologically relevant nutrient and oxygen gradients [104]. The growth kinetics analysis revealed a characteristic sigmoidal pattern well-described by the Gompertz model (R^2^ = 0.998), with an estimated doubling time of 2.1 days [105]. This rapid growth rate underscores the aggressive nature of this TNBC cell line and highlights the importance of 3D models in recapitulating in vivo tumor dynamics [106]. The IC50 values in 3D cultures differed from those in 2D, with 3D spheroids showing higher IC50 for VCA (500 ng/mL vs. 30 ng/mL) but lower for cisplatin (90 μg/mL vs. 130 μg/mL). The higher IC50 for VCA in 3D cultures aligns with typical expectations, as the three-dimensional structure can impede drug penetration due to increased cell–cell contacts and matrix interactions. However, the lower IC50 observed for cisplatin in 3D cultures is intriguing and may be attributed to several factors. First, the hypoxic conditions often present in spheroid cores can enhance cisplatin’s cytotoxicity through the generation of reactive oxygen species and DNA damage. Second, the altered expression of drug transporters in 3D cultures might affect cisplatin accumulation differently than VCA. Additionally, the different cell viability assays used for 2D (MTT) and 3D (CellTiter-Glo) cultures might contribute to these variations, as they measure different aspects of cellular metabolism. The MTT assay measures mitochondrial activity, while CellTiter-Glo measures ATP levels, which might be differently affected under 3D culture conditions. The combination index analysis revealed more complex, concentration-dependent interactions between VCA and cisplatin in 3D cultures, transitioning from synergism at higher concentrations to antagonism at lower concentrations. This complexity underscores the importance of careful dose optimization for potential clinical applications [107]. The pattern of cell death in treated spheroids, progressing from the periphery inward with higher doses, is consistent with limited drug penetration into the spheroid core [108]. The more pronounced effects seen with combination treatments suggest that VCA and cisplatin may work synergistically to overcome these penetration barriers [109].

Gene expression analysis revealed more pronounced effects of VCA and cisplatin treatment in 3D spheroids compared to 2D cultures. At IC50 concentrations, Bax upregulation was 4.1-fold in 3D vs. 3.2-fold in 2D, MMP-9 downregulation was 0.2-fold in 3D vs. 0.3-fold in 2D, and E-cadherin upregulation was 3.5-fold in 3D vs. 2.8-fold in 2D (all *p* < 0.001). These findings suggest stronger pro-apoptotic, anti-metastatic, and EMT-inhibiting effects in a 3D environment than predicted by 2D studies [110]. The PCA of our 3D culture data provided a comprehensive view of the cellular response to treatments. The clear separation of treatment groups in the PCA plot underscores the distinct cellular responses elicited by VCA and cisplatin, both alone and in combination. The positioning of combination treatments, particularly the high-dose combination, furthest from the control group corroborates our findings of synergistic effects [111].

The enhanced effects observed in 3D cultures compared to 2D can be attributed to several factors. Three-dimensional spheroids better recapitulate the oxygen and nutrient gradients found in tumors, which can affect drug penetration and efficacy [112]. Additionally, 3D cultures allow for more natural cell–cell and cell–matrix interactions, which can influence gene expression and drug response [113]. These factors contribute to making 3D models more predictive of in vivo responses than traditional 2D cultures. The consistency in synergistic effects across both 2D and 3D models strengthens the rationale for further investigation of this combination therapy. Nevertheless, this study has several limitations. Our in vitro models cannot fully represent in vivo conditions, including the tumor microenvironment, immune system interactions, and systemic effects. Additionally, optimal dosing for clinical application remains to be determined through pharmacokinetic and pharmacodynamic studies.

This study suggests that VCA and cisplatin have favorable effects in combating MDA-MB-231 cells, which are used to model TNBC. The combination therapy showed a greater effect on cell viability, apoptosis, cell cycle arrest, migration, and EMT reversal than either agent alone. Some of these effects were mediated through the regulation of apoptotic proteins, cell cycle regulators, EMT markers, and MMPs, implying that the combination therapy may be useful in antitumor and anti-metastatic activities. Based on these results, VCA may be suggested to be used together with cisplatin-based therapy in patients with TNBC for the enhancement of treatment outcomes, particularly concerning the questions of drug resistance and metastasis. However, it is crucial to emphasize that while our results are promising, they are preliminary and require further validation. Specifically, future in vivo studies utilizing both xenograft models and patient-derived xenografts (PDXs) will be essential to validate our findings. Xenograft models using MDA-MB-231 cells would provide a direct continuation of our current work and allow for the evaluation of the VCA–cisplatin combination’s efficacy in a complete biological system [114]. Furthermore, PDX models, which better preserve the heterogeneity in and molecular characteristics of the original tumor [115], would offer more clinically relevant insights into the treatment’s potential effectiveness across different TNBC subtypes. These models would also enable the assessment of important parameters that cannot be evaluated in vitro, such as drug biodistribution, optimal dosing schedules, potential toxicities, and effects on the tumor microenvironment. Additionally, studying the treatment’s impact on metastasis formation in these models would be particularly valuable given TNBC’s high metastatic potential.

The findings from this study have several important clinical implications for TNBC treatment [116]. First, our demonstration of synergistic effects between VCA and cisplatin at relatively low concentrations suggests the potential for dose reduction in cisplatin in combination therapy, which could help minimize cisplatin-associated toxicities while maintaining therapeutic efficacy [117]. This is particularly relevant for TNBC patients who often experience severe side effects from conventional chemotherapy. Second, the enhanced anti-metastatic effects observed in both 2D and 3D models suggest that this combination might be particularly beneficial for patients with aggressive or metastatic TNBC [118]. Third, the differential effects observed between 2D and 3D models highlight the importance of careful dose optimization in future clinical applications. Looking ahead, several key steps would be necessary to translate these findings to clinical practice: (1) comprehensive in vivo studies to confirm safety and efficacy, (2) careful optimization of dosing schedules and ratios, (3) development of appropriate delivery systems, and (4) identification of potential biomarkers for patient stratification [116,118]. Future clinical trials might focus initially on TNBC patients who have shown partial response or resistance to conventional cisplatin therapy, as these patients might particularly benefit from the synergistic effects observed in our study.

In conclusion, this study provides substantial preliminary evidence for the synergistic effects of VCA and cisplatin in TNBC treatment, specifically in preclinical in vitro models, demonstrating enhanced cytotoxicity, anti-metastatic potential, and multifaceted molecular impacts. While these effects were consistent across 2D and 3D models, further validation is essential. However, in vitro systems cannot fully replicate the complexity of tumor microenvironments or patient heterogeneity. Future research should focus on comparative analyses of cancer and normal breast cell responses in both 2D and 3D models to better predict therapeutic windows and refine dosing strategies. Developing matched normal–cancer 3D culture systems would further enhance these efforts.

Future studies should also evaluate the selective toxicity profile of the VCA–cisplatin combination through in vivo research and biomarker identification for treatment response. Optimizing dosing strategies is critical for maximizing efficacy and minimizing toxicity. Particularly, the observed synergistic effects suggest the potential for reducing cisplatin dosage while maintaining therapeutic efficacy, which could significantly decrease cisplatin-associated toxicities that often limit its clinical use. This strategy could be particularly beneficial for TNBC patients who often require aggressive chemotherapy. Given the molecular heterogeneity in TNBC, validation studies should incorporate diverse TNBC models representing various subtypes, as responses may differ significantly. A comprehensive validation approach would help identify the patient populations most likely to benefit from this therapy. While the VCA–cisplatin combination represents a promising direction, extensive animal studies and carefully designed clinical trials are necessary before clinical application can be realized.

## 4. Materials and Methods

### 4.1. Purification of VCA

Korean mistletoe was collected from oak trees in Kangwon-do, Korea, and its botanical identity was confirmed by Professor Jon-Suk Lee, Seoul Women’s University, Korea. Lectin was purified as previously described using SP Sephadex C-50 (Cat# GE17-0240-01, Sigma, St. Louis, MO, USA) and asialofetuin–Sepharose 4B (Cat# A4781, Sigma). Ultrafiltration (Cat# PLCGC10205, Millipore EMD, Billerica, MA, USA) was performed for concentration [57]. The protein concentration was determined using the Bio-Rad protein assay kit (Cat# 5000001, Bio-Rad, Hercules, CA, USA). The molecular mass and purity of lectin were analyzed by sodium dodecyl sulfate–polyacrylamide gel electrophoresis (SDS-PAGE) with a 12% polyacrylamide resolving gel and a 4% acrylamide stacking gel. Samples were treated with or without 1% 2-mercaptoethanol (Cat# M3148, Sigma), electrophoresed at 100 V for 1 h, and stained with Coomassie Brilliant Blue R-250 (Cat# 1.12553, Sigma) for 1 h.

### 4.2. Two-Dimensional Cell Culture

Human TNBC cell lines, MDA-MB-231 (passage number 14) and BT-549 (passage number 10), were cultured in Dulbecco’s Modified Eagle Medium (DMEM, Cat# DMEM/F-12-11320033, Gibco, Carlsbad, CA, USA) supplemented with 10% fetal bovine serum (FBS, Cat# 16000044, Gibco), 100 U/mL penicillin, and 100 μg/mL streptomycin (Cat# 15140122, Gibco). The human normal breast epithelial cell line MCF-10A (passage number 16) was maintained in DMEM/F-12 (Cat# 11320033, Gibco) supplemented with 5% horse serum (Cat# 16050122, Gibco), 20 ng/mL human epidermal growth factor (EGF, Cat# PHG0311, Thermo Fisher Scientific, Waltham, MA, USA), 0.5 μg/mL hydrocortisone (Cat# H0888, Sigma), 10 μg/mL insulin (Cat# I1882, Sigma), 100 U/mL penicillin, and 100 μg/mL streptomycin (Cat# 15140122, Gibco). Cells were incubated at 37 °C in a humidified atmosphere containing 5% CO_2_. Mycoplasma contamination of the cell line was routinely tested using the MycoAlert™ Mycoplasma Detection Kit (Lonza, Cat# LT07-318, Basel, Switzerland), and no contamination was observed during the course of this study. Cisplatin was purchased from Sigma (Cat# 1134357) and dissolved in dimethyl sulfoxide (DMSO) to prepare stock solutions. For all experiments, untreated cells maintained in complete medium served as negative controls. To ensure experimental reproducibility, all experiments were performed with at least three independent biological replicates using different passage numbers. For each biological replicate, technical triplicates were included. Cell viability assays were performed by two independent researchers in a blinded manner. Quality control measures included (1) regular authentication of cell lines using short tandem repeat (STR) profiling, (2) standardization of cell seeding density and incubation times, (3) inclusion of appropriate positive and negative controls in each experiment, and (4) consistent criteria for data inclusion/exclusion. Only experiments with control values within a 10% standard deviation were included in the final analysis.

### 4.3. Two-Dimensional Cell Viability Assay

For 2D cell culture experiments, cells were treated with VCA at concentrations ranging from 1 to 500 ng/mL (1, 5, 10, 50, 100, and 500 ng/mL) or cisplatin at concentrations ranging from 1 to 200 μg/mL (1, 5, 10, 100, 150, and 200 μg/mL). For combination treatments, VCA was used at IC20 (4 ng/mL) or IC50 (30 ng/mL) concentrations in combination with cisplatin. These concentrations were determined based on initial dose–response studies. For normal cell toxicity studies, MCF-10A cells were treated with the same concentration ranges of VCA and cisplatin. The effect of VCA and cisplatin on the viability of MDA-MB-231 cells was assessed using 3-(4,5-dimethylthiazol-2-yl)-2,5-diphenyltetrazolium bromide (MTT, Cat# M5655, Sigma) and crystal violet assays. For the MTT assay, cells were seeded in 96-well plates at a density of 5 × 10^3^ cells per well and allowed to attach overnight. Following treatment with VCA or cisplatin or both for 24, 48, and 72 h, 20 μL of MTT solution (5 mg/mL in PBS) was added to each well and incubated for an additional 4 h at 37 °C. The formazan crystals were dissolved in 150 μL of DMSO, and the absorbance was measured at 570 nm using a microplate reader (Sunrise^®^, Tecan, Switzerland). For the assessment of cytotoxicity in MCF-10A and BT-549 cells, a water-soluble tetrazolium salt (WST-1, Cat# 05015944001, Sigma) assay was performed. Cells were seeded in 96-well plates at a density of 5 × 10^3^ cells per well and allowed to attach overnight. After 48 h treatment with VCA or cisplatin, 10 μL of WST-1 reagent was added to each well and incubated for 2 h at 37 °C. The absorbance was measured at 450 nm using the same microplate reader.

### 4.4. Apoptosis Assay

Apoptotic cells were quantified using the Muse Annexin V and Dead Cell Kit (Cat# KSU MCH100105, Millipore EMD) according to the manufacturer’s protocol. Cells were treated with VCA, cisplatin, or their combination for 48 h. The cell suspension was mixed with Muse Annexin V and Dead Cell Kit reagent and incubated at room temperature for 20 min in the dark.

Apoptotic cells were quantified and analyzed using the Muse Cell Analyzer (Millipore EMD). Live/dead cell staining was conducted using AO and PI dyes. Treated cells were washed with PBS and incubated with a mixture of AO (10 μg/mL) and PI (10 μg/mL) in a serum-free medium for 15 min at 37 °C in the dark. Cells were observed under a fluorescence microscope (Nikon, Tokyo, Japan) using a dual filter set for green (AO) and red (PI) fluorescence. For quantification, at least 10 random fields per condition were captured and analyzed using ImageJ software (NIH, Bethesda, MD, USA). The gating strategy was standardized across all samples as follows: lower left quadrant (Annexin V-negative/7-AAD-negative): viable cells, lower right quadrant (Annexin V-positive/7-AAD-negative): early-apoptotic cells, upper right quadrant (Annexin V-positive/7-AAD-positive): late-apoptotic/dead cells, and upper left quadrant (Annexin V-negative/7-AAD-positive): nuclear debris. The quadrant positions were set using unstained and single-stained controls, and these positions were maintained consistently across all experimental samples. The percentages of viable and dead cells were calculated based on the total fluorescence intensities of AO (green) and PI (red), with careful consideration of background correction and threshold settings.

### 4.5. Cell Cycle Analysis

MDA-MB-231 cells (2 × 10^5^ cells per 100 mm^2^ dish) were treated with VCA, cisplatin, or their combination for 48 h. Cells were harvested, washed with PBS (pH 7.4), and fixed in 70% ethanol overnight at −20 °C. Fixed cells were washed and stained with propidium iodide (PI) solution containing RNase A. The cell cycle distribution was analyzed using the Muse Cell Cycle Kit and Muse Cell Analyzer (Cat# SKU MCH100106, Millipore EMD) according to the manufacturer’s protocol. The percentages of cells in the G0/G1, S, and G2/M phases were determined e Muse Cell Cycle Software version 1.5.0.0 (Millipore EMD).

### 4.6. Migration and Invasion Assay

The migratory and invasive potential of MDA-MB-231 cells following treatment with VCA and cisplatin was evaluated using Transwell chambers (Cat# CLS3414, Corning, Lake Placid, NY, USA) with 8 μm pore size polycarbonate membranes. For the migration assay, cells were seeded in the upper chamber in a serum-free medium, while the lower chamber contained a medium supplemented with 10% FBS as a chemoattractant. After 12 h of incubation, the non-migrated cells on the upper surface of the membrane were removed with a cotton swab, and the migrated cells on the lower surface were fixed with 4% paraformaldehyde and stained with 0.1% crystal violet. For the invasion assay, the upper chamber was coated with Matrigel (Cat# CLS354234, Corning) to mimic the extracellular matrix. The invasion assay was performed similarly to the migration assay but with a 24 h incubation period. The number of migrated and invaded cells was counted in five randomly selected fields under a light microscope (Olympus, Tokyo, Japan) at 100× magnification. The results were expressed as the average number of cells per field.

### 4.7. Wound Healing Assay

To assess the effects of VCA and cisplatin on the migratory potential of MDA-MB-231 cells, a wound healing assay was performed. Cells were seeded in 6-well plates at a density of 5 × 10^5^ cells/well and grown to 90% confluence. A straight scratch was created in the cell monolayer using a sterile 200 μL pipette tip. The cells were then washed twice with PBS to remove debris and treated with VCA, cisplatin, or a combination of both in serum-free medium. Untreated cells served as a control. Wound closure was monitored and photographed at 0, 12, and 24 h using an inverted microscope (Olympus, Japan) at 100× magnification. For quantification, the wound area was manually traced and measured using the freehand selection tool in ImageJ software (National Institutes of Health, NIH, Bethesda, MD, USA). The percentage of wound closure was calculated using the formula: Wound closure (%) = [(Initial area − Final area)/Initial area] × 100. Multiple fields were analyzed for each condition, and the experiment was performed in triplicate. The data were presented as mean ± standard deviation (SD).

### 4.8. Synergy Analysis

To evaluate the synergistic effects of VCA and cisplatin combination treatments, we employed the Excess Over Highest Single Agent (EOHSA) method. EOHSA was calculated for migration, invasion, and wound healing assays using the following formula: EOHSA = Effect of combination − Effect of most potent single agent. Negative EOHSA values indicate synergy, demonstrating that the combination treatment is more effective than the most potent single agent. Additionally, we calculated the combination index (CI) using the Chou–Talalay method to further quantify the synergistic effects. CI analysis was performed using CompuSyn software (Version 1.0, ComboSyn Inc., Paramus, NJ, USA) based on the Chou–Talalay method. The CI values were calculated using the formula CI = (D)1/(Dx)1 + (D)2/(Dx)2, where (D)1 and (D)2 are the doses of drugs 1 and 2 that in combination produce x% effect, and (Dx)1 and (Dx)2 are the doses of drugs 1 and 2 that alone produce the same effect. A CI value of <1 represents synergy, a value of 1 indicates additive effects, and a value of >1 indicates antagonism. The fractional effect (Fa) was calculated as 1 − (treated/control) for each treatment condition. CI and fractional effect (Fa) values were plotted to generate Fa-CI plots. To determine optimal concentrations for combination studies, cells were first treated with serial dilutions of VCA (1–500 ng/mL) or cisplatin (1–200 μg/mL) as single agents for 24–72 h. From these dose–response curves, IC20 and IC50 values were calculated using GraphPad Prism software (Version 9.5.1, GraphPad Software, Boston, MA, USA) by fitting the normalized response data to a four-parameter logistic curve. The obtained IC values (4 ng/mL and 30 ng/mL for VCA; 7 μg/mL and 130 μg/mL for cisplatin) were then used to design combination experiments. For the systematic evaluation of drug interactions, a combination matrix was designed, where fixed concentrations of VCA (IC20 and IC50) were tested against a range of cisplatin concentrations (1– 200 μg/mL) in a non-constant ratio approach.

### 4.9. Western Blot Analysis

The expression of apoptosis-, metastasis-, and EMT-related proteins was analyzed by Western blotting. MDA-MB-231 cells were treated with VCA and cisplatin for 48 h, and total protein was extracted using RIPA lysis buffer (Cat# 89901, Thermo Fisher Scientific) containing protease and phosphatase inhibitors. Protein concentrations were determined using the BCA Protein Assay Kit (Cat# 23227, Thermo Fisher Scientific). Equal amounts of protein (30 μg) were separated by SDS-PAGE and transferred onto polyvinylidene difluoride (PVDF) membranes (Cat# IPVH00010, Millipore EMD). Membranes were blocked with 5% non-fat milk in Tris-buffered saline containing 0.1% Tween-20 (TBST) for 1 h at room temperature and then incubated with primary antibodies overnight at 4 °C. The following primary antibodies were used (all from Cell Signaling Technology, Danvers, MA, USA), each diluted 1:1000 in TBST: anti-Bcl-2 (Cat# 4223), anti-Bax (Cat# 5023), anti-cleaved caspase-3 (Cat# 9964), anti-cleaved PARP(Cat#5625), anti-caspase-3 (Cat# 14220), anti-MMP-2 (Cat# 89809), anti-MMP-9 (Cat# 13667), anti-N-cadherin (Cat# 13116), anti-E-cadherin (Cat# 3195), anti-vimentin (Cat# 5741), anti-β-catenin (Cat#8480), anti-Snail (Cat# 3879), anti-Slug (Cat# 9585), and anti-β-actin (Cat# 4970). After washing with TBST, the membranes were incubated with horseradish peroxidase (HRP)-conjugated secondary antibodies (Cat#7074) for 1 h at room temperature. Protein bands were visualized using an enhanced chemiluminescence (ECL) detection system (Cat# 28980926, GE Healthcare, Chicago, IL, USA). Protein expression levels were quantified by densitometric analysis using ImageJ software (NIH). Band intensities were normalized to β-actin as an internal loading control, with the control sample set as 1.0 for relative quantification. All quantification was performed on at least three independent experiments.

### 4.10. Quantitative Real-Time Polymerase Chain Reaction (PCR)

Total RNA was extracted from treated cells using TRIzol reagent (Cat# 15596018, Invitrogen, Carlsbad, CA, USA) according to the manufacturer’s instructions. cDNA was synthesized using the SuperScript III First-Strand Synthesis System (Cat# 18080051, Invitrogen). qPCR was performed using SYBR Green PCR Master Mix (Cat# SKU4367659, Applied Biosystems, Foster City, CA, USA) on a StepOnePlus Real-Time PCR System (Cat# MR2145, Applied Biosystems). Each reaction contained 5 μL of 2× SYBR Green PCR Master Mix, 0.5 μL of forward and reverse primers (10 μM each), 2 μL of diluted cDNA, and 2 μL of nuclease-free water, totaling 10 μL. The PCR cycling conditions were as follows: initial denaturation at 95 °C for 10 min, followed by 40 cycles of denaturation at 95 °C for 15 s, and annealing/extension at 60 °C for 1 min. A melting curve analysis was performed at the end of each run to confirm amplification specificity. We used the following primer sequences: Bcl-2: forward 5′-GGTGGGGTCATGTGTGTGG-3′, reverse 5′-CGGTTCAGGTACTCAGTCATCC-3′; Bax: forward 5′-CCCGAGAGGTCTTTTTCCGAG-3′, reverse 5′-CCAGCCCATGATGGTTCTGAT-3′; caspase-3: forward 5′-CATGGAAGCGAATCAATGGACT-3′, reverse 5′-CTGTACCAGACCGAGATGTCA-3′; PARP: forward 5′-CGGAGTCTTCGGATAAGCTCT-3′, reverse 5′-TTTCCATCAAACATGGGCGAC-3′; MMP-2: forward 5′-TACAGGATCATTGGCTACACACC-3′, reverse 5′-GGTCACATCGCTCCAGACT-3′; MMP-9: forward 5′-TGTACCGCTATGGTTACACTCG-3′, reverse 5′-GGCAGGGACAGTTGCTTCT-3′; E-cadherin: forward 5′-CGAGAGCTACACGTTCACGG-3′, reverse 5′-GGGTGTCGAGGGAAAAATAGG-3′; N-cadherin: forward 5′-TCAGGCGTCTGTAGAGGCTT-3′, reverse 5′-ATGCACATCCTTCGATAAGACTG-3′; vimentin: forward 5′-GACGCCATCAACACCGAGTT-3′, reverse 5′-CTTTGTCGTTGGTTAGCTGGT-3′; β-catenin: forward 5′-AAAGCGGCTGTTAGTCACTGG-3′, reverse 5′-CGAGTCATTGCATACTGTCCAT-3′; Snail: forward 5′-TCGGAAGCCTAACTACAGCGA-3′, reverse 5′-AGATGAGCATTGGCAGCGAG-3′; Slug: forward 5′-CGAACTGGACACACATACAGTG-3′, reverse 5′-CTGAGGATCTCTGGTTGTGGT-3′; ZEB1: forward 5′-TTCAAACCCATAGTGGTTGCT-3′, reverse 5′-TGGGAGATACCAAACCAACTG-3′ Twist: forward 5′-GGAGTCCGCAGTCTTACGAG-3′, reverse 5′-TCTGGAGGACCTGGTAGAGG-3′; and GAPDH: forward 5′-GGAGCGAGATCCCTCCAAAAT-3′, reverse 5′-GGCTGTTGTCATACTTCTCATGG-3′. GAPDH was used as an internal control for normalization. All primers were validated for specificity and efficiency prior to use in this study. The relative gene expression was calculated using the 2^−ΔΔCt^ method. The Ct values of target genes were normalized to those of GAPDH to obtain ΔCt values, and then the ΔCt values of the treated samples were normalized to the ΔCt of the untreated control to calculate ΔΔCt. The fold change in gene expression was calculated as 2^−ΔΔCt^. All experiments were performed in triplicate, and the results were presented as mean ± standard deviation (SD).

### 4.11. Heatmap Analysis

Gene expression data from real-time PCR experiments were used to generate a heatmap. Log2-fold changes relative to the control were calculated for each gene across all treatment conditions. The heatmap was created using Microsoft Excel (LTSC Professional Plus 2021) with conditional formatting. Genes were clustered based on similarities in expression patterns across treatments. The color scale was set to diverge from blue (downregulation) to white (no change) to red (upregulation), with the intensity of the color representing the magnitude of change.

### 4.12. Correlation Analysis

To investigate the relationship between protein and mRNA expression levels, as well as 3D spheroid parameters (invasion area, spheroid size, and viability), correlation analyses were performed. Relative protein expression data (from Western blots), relative mRNA expression data (from qPCR), and 3D spheroid measurement data were analyzed using GraphPad Prism version 9.5.1 (GraphPad Software). Linear regression with Pearson’s correlation coefficient was employed based on several criteria: (1) the normality of the data distribution was confirmed using the Shapiro–Wilk test (*p* > 0.05), (2) visual inspection of scatter plots demonstrated clear linear relationships without evident non-linear patterns, and (3) no significant outliers were detected in the dataset. Pearson’s correlation coefficients (r) and their statistical significance (*p*-values) were calculated. A *p*-value < 0.05 was considered statistically significant.

### 4.13. Enzyme-Linked Immunosorbent Assay (ELISA)

Vascular endothelial growth factor (VEGF) and Hypoxia-Inducible Factor 1-alpha (HIF-1α) levels in cell culture supernatants were measured using the Human VEGF Quantikine ELISA Kit (Cat# DVE00) and Human HIF-1α DuoSet ELISA (Cat# DYC1935-2, R&D Systems, Minneapolis, MN, USA), respectively. All assays were performed according to the manufacturer’s instructions. Briefly, samples were incubated in antibody-coated microplates, followed by the addition of enzyme-linked detection antibodies. After washing, a substrate solution was added, and the resulting color change was measured spectrophotometrically. Absorbance was measured at 450 nm using a microplate reader (Sunrise^®^, Tecan). Standard curves were generated to quantify VEGF and HIF-1α concentrations in the samples.

### 4.14. Principal Component Analysis (PCA)

A PCA was performed using Microsoft Excel (LTSC Professional Plus 2021) with the Data Analysis ToolPak add-in. The data matrix included parameters such as cell viability, apoptosis rates, cell cycle distribution percentages, migration and invasion rates, wound healing percentages, and gene expression levels for each treatment condition. Data were standardized by mean-centering and scaling to unit variance for each parameter. The correlation matrix was calculated, followed by eigenvalue decomposition. This process integrated data from multiple assays into a single analysis, providing a comprehensive view of treatment effects. The first two principal components, explaining the majority of the variance, were used to create a two-dimensional scatter plot.

### 4.15. Three-Dimensional Cell Culture and Viability Assay

Magnetic levitation using the Bio-Assembler Kit (Cat# 657840, Nano3D Biosciences, Houston, TX, USA) was employed to create 3D cultures, as previously reported [119]. To minimize experimental variability, all experiments were performed with at least three independent biological replicates using different cell passages. A standardized seeding density of 5000 cells per well was maintained across all experiments, and only spheroids with an initial diameter variation of less than 10% were included in analyses. Environmental parameters were strictly controlled (temperature: 37 ± 0.5 °C, CO_2_: 5 ± 0.2%, humidity: >95%). In short, NanoShuttle^TM^ was added to flasks of MDA-MB231 cells grown in 2D at 80% confluence and incubated overnight. Cells were detached with trypsin and resuspended in media, and the cell suspension was added to 24- (Cat# 3473) and 96-well (Cat# CLS3474) ultra-low attachment plates (Corning, Tewksbury, MA, USA). A magnetic driver of 24 and 96 neodymium magnets (field strength = 50 G) and a plastic lid insert were placed atop (for 24-well) or below (for 96-well) the plate to levitate the cells to the air–liquid interface. Cells were then incubated at 37 °C in a humidified, 5% CO_2_ atmosphere overnight in an incubator. Spheroid formation and growth were monitored daily with phase-contrast microscopy to maintain consistency. Quality control measures included regular assessment of spheroid morphology, size, and viability. The viability of 3D cultures was evaluated with the CellTiter-Glo 3D Cell Viability Assay (Cat# G9681, Promega, Madison, WI, USA) following the manufacturer’s instructions. Luminescence was measured using a microplate reader (Tecan Infinite M200). Dose–response curves were generated for VCA and cisplatin individually and in combination.

### 4.16. Three-Dimensional Spheroid Morphological Analysis

Brightfield images of MDA-MB-231 spheroids captured over 8 days were analyzed using ImageJ software (NIH, Bethesda, MD, USA). The outline of each spheroid was manually traced, and the “Analyze Particles” function was used to measure circularity and solidity. Circularity is calculated as 4π (area/perimeter^2^), with a value of 1.0 indicating a perfect circle. Solidity is the ratio of the area to the convex hull area, where higher values indicate a more compact shape. The spheroid diameter was measured using the straight-line tool in ImageJ. At least 20 spheroids were analyzed for each time point, and the experiment was repeated three independent times. Immediately after seeding, cells began to aggregate, forming loose clusters by day 1. The spheroids grew steadily, reaching a mean diameter of 355 ± 15 μm by day 2, 552 ± 23 μm by day 5, and 708 ± 30 μm by day 8 (Figure 13). We further analyzed spheroid morphology by quantifying circularity and solidity. Circularity, which indicates how closely the spheroid shape approaches a perfect circle, increased from 0.72 ± 0.05 on day 0 to 0.89 ± 0.03 by day 2 and continued to increase slightly to 0.91 ± 0.02 by day 8. Solidity, reflecting the compactness of the spheroid, showed a similar trend, increasing from 0.84 ± 0.04 on day 0 to 0.95 ± 0.02 by day 2 and reaching 0.97 ± 0.01 by day 8. These results indicate that MDA-MB-231 cells develop more circular and compact spheroids over time, with the most significant changes observed within the first 48 h of culture.

### 4.17. Three-Dimensional Spheroid Growth Kinetics

To analyze growth kinetics, the spheroid volume was calculated assuming a spherical shape (V = 4/3πr^3^, where r is the radius). The growth curve was fitted to a Gompertz model using the following equation: V(t) = V0 × exp((A/α) × (1 − exp(−αt))), where V(t) is the volume at time t, V0 is the initial volume, A is the growth rate, and α is the growth deceleration factor. The instantaneous growth rate was derived from the Gompertz function’s first derivative: dV/dt = A × V(t) × exp(−αt). The growth kinetics of MDA-MB-231 spheroids were also monitored over 8 days. The spheroid volume increased significantly from 0 to 1.86 ± 0.06 × 10^8^ μm^3^ (mean ± SD) over this period (*p* < 0.001, one-way ANOVA). The growth pattern closely followed a Gompertz model (R^2^ = 0.998), with fitted parameters V0 = 1.2 × 10^6^ μm^3^ (95% CI: 0.9–1.5 × 10^6^), A = 0.76 day-1 (95% CI: 0.68–0.84) and α = 0.22 day-1 (95% CI: 0.18–0.26). The instantaneous growth rate increased from 11.7 ± 0.5 × 10^6^ μm^3^/day on day 2 to 32.7 ± 1.2 × 10^6^ μm^3^/day on day 8. The estimated doubling time was 2.1 ± 0.2 days.

### 4.18. Real-Time PCR Analysis of 3D Spheroids

After 8 days of spheroid formation, they were treated with 143 ng/mL VCA, 30 μg/mL cisplatin, their combination, 500 ng/mL VCA, 90 μg/mL cisplatin, or their combination for 72 h. Total RNA was extracted from the spheroids using the RNeasy 3D Cell Mini Kit (Cat# 74104, Qiagen) according to the manufacturer’s instructions. cDNA synthesis and real-time PCR were performed as described for 2D cultures. We used the following primers: Bax (forward 5′-CCCGAGAGGTCTTTTTCCGAG-3′, reverse 5′-CCAGCCCATGATGGTTCTGAT-3′), MMP-9 (forward 5′-TGTACCGCTATGGTTACACTCG-3′, reverse 5′-GGCAGGGACAGTTGCTTCT-3′), E-cadherin (forward 5′-CGAGAGCTACACGTTCACGG-3′, reverse 5′-GGGTGTCGAGGGAAAAATAGG-3′), and GAPDH (forward 5′-GGAGCGAGATCCCTCCAAAAT-3′, reverse 5′-GGCTGTTGTCATACTTCTCATGG-3′). Gene expression was normalized to GAPDH and presented as fold change relative to the control, calculated by the 2^−ΔΔCt^ method.

### 4.19. Three-Dimensional Spheroid Invasion Assay

Spheroids were embedded in a collagen matrix (2 mg/mL) and treated with VCA, cisplatin, or their combinations. Spheroid growth and invasion were monitored for 4 days using brightfield microscopy. Images were captured on 0 and 4 days using an Olympus IX73 inverted microscope. For quantification of invasion, both the total area occupied by the invading cells and the core spheroid area were measured using ImageJ software (NIH, Bethesda, MD, USA). The invasion area was determined by subtracting the initial spheroid area (day 0) from the total area encompassing invasive structures (day 4).

### 4.20. PCA of 3D Culture Data

To visualize the overall effects of VCA and cisplatin treatments on MDA-MB-231 cells in 3D culture, we performed a PCA using cell viability data, live/dead cell data, invasion data, and gene expression data for Bax, MMP-9, and E-cadherin. The analysis was performed using Microsoft Excel with the Data Analysis ToolPak as described earlier. A two-dimensional scatter plot was generated using the first two principal components.

### 4.21. Statistical Analysis

Statistical analyses were performed using GraphPad Prism version 9.5.1 (GraphPad Software). All experiments were performed with at least three independent biological replicates (n ≥ 3), and data are presented as mean ± SD with 95% confidence intervals. For cell viability assays (MTT and crystal violet), one-way ANOVA followed by Dunnett’s post hoc test was used to compare treatment groups to the control. For drug combinations, two-way ANOVA followed by Tukey’s post hoc test was used. For all other experiments, one-way ANOVA followed by Tukey’s post hoc test was used to compare multiple groups. The normality of the data distribution was verified using the Shapiro–Wilk test before applying parametric tests. For non-normally distributed data, the Kruskal–Wallis test followed by Dunn’s post hoc test was used. For the live/dead cell assay, significance for viable and dead cells was analyzed separately. For Western blot quantification, statistical analysis was performed on densitometric data from three independent experiments. For flow cytometry analyses, at least 10,000 events were analyzed per sample. For all analyses, a *p*-value < 0.05 was considered statistically significant.

## Data Availability

Data are contained within the article.
